# Selection and characterization of human scFvs targeting the SARS-CoV-2 nucleocapsid protein isolated from antibody libraries of COVID-19 patients

**DOI:** 10.1038/s41598-024-66558-0

**Published:** 2024-07-09

**Authors:** Simonetta Lisi, Francesca Malerba, Paola Quaranta, Rita Florio, Ottavia Vitaloni, Elisa Monaca, Bruno Bruni Ercole, Angela Rachel Bitonti, Olga del Perugia, Marianna Mignanelli, Paola Perrera, Raffaele Sabbatella, Francesco Raimondi, Carmen Rita Piazza, Anna Moles, Caterina Alfano, Mauro Pistello, Antonino Cattaneo

**Affiliations:** 1 Bio@SNS Laboratory, Scuola Normale Superiore, 56126 Pisa, Italy; 2 Fondazione EBRI (European Brain Research Institute) Rita Levi-Montalcini, 00161 Rome, Italy; 3 Structural Biology and Biophysics Unit, Fondazione Ri.MED, 90133 Palermo, Italy; 4 Retrovirus Centre, Department of Translational Research, University of Pisa, 56126 Pisa, Italy; 5 Virology Operative Unit, Pisa University Hospital, 56124 Pisa, Italy; 6 Department of Medical Biotechnologies, University of Siena, 53100 Siena, Italy; 7 Genomnia Srl, 20091 Bresso, MI Italy; 8 Institute of Biochemistry and Cell Biology, CNR, 80131 Napoli, Italy

**Keywords:** Biochemistry, Biotechnology, Immunology, Molecular biology

## Abstract

In 2019, the novel SARS-CoV-2 coronavirus emerged in China, causing the pneumonia named COVID-19. At the beginning, all research efforts were focused on the spike (S) glycoprotein. However, it became evident that the nucleocapsid (N) protein is pivotal in viral replication, genome packaging and evasion of the immune system, is highly immunogenic, which makes it another compelling target for antibody development alongside the spike protein. This study focused on the construction of single chain fragments variable (scFvs) libraries from SARS-CoV-2-infected patients to establish a valuable, immortalized and extensive antibodies source. We used the Intracellular Antibody Capture Technology to select a panel of scFvs against the SARS-CoV-2 N protein. The whole panel of scFv was expressed and characterized both as intrabodies and recombinant proteins. ScFvs were then divided into 2 subgroups: those that exhibited high binding activity to N protein when expressed in yeast or in mammalian cells as intrabodies, and those purified as recombinant proteins, displaying affinity for recombinant N protein in the nanomolar range. This panel of scFvs against the N protein represents a novel platform for research and potential diagnostic applications.

## Introduction

In 2019, the human Severe Acute Respiratory Syndrome Coronavirus (SARS-CoV-2) was isolated in China from bronchoalveolar lavage fluid samples of patients. It was then defined as the etiological cause of a pneumonia outbreak, named COVID-19^1,2^.

SARS-CoV-2 belongs to the Coronaviridae family, genus *Betacoronavirus* (β-CoV)^3^*.* The viral genome consists of a positive-sense single-stranded RNA (+ ssRNA) of approximately 29.9 kb and is characterized by a high sequence identity with some SARS-related coronaviruses found in bats, as well as with SARS-CoV and MERS-CoV genomes^2,4^. The SARS-CoV-2 genome comprises 14 open reading frames (ORFs). Those located in the 5’-end encode for 16 non-structural proteins (nsps), which are functionally involved in viral replication and transcription. Conversely, ORFs at the 3’ terminus encode accessory proteins and four structural proteins, namely spike (S) glycoprotein, nucleocapsid (N) protein, membrane (M) protein and envelope (E) protein^5,6^.

The S glycoprotein allows the virus to infect target cells via its receptor binding domain (RBD), which is responsible for the binding between the viral particle and the host cell receptor, i.e. the angiotensin-2-converting enzyme (ACE2)^7,8^. After this first interaction, the S protein undergoes a fundamental conformational transition. This change facilitates the fusion of the viral particle with the host cell membrane, initiating the subsequent steps of viral replication, which are accomplished by exploiting the cellular translation machinery^9^. In the context of viral replication, the N protein performs important key functions. It binds and packages the viral genome and contributes to new virions assembly^10,11^. Moreover, the structural N protein contributes to SARS-CoV-2 immune escape acting as a viral suppressor of RNA interference (RNAi), a cell-intrinsic antiviral immune defence mechanism, and suppressing IFN activation and IFN-mediated antiviral pathway^12,13^.

In addition to their functional roles, the N and S proteins are the major immunogenic proteins of coronaviruses, as demonstrated from serological analyses performed on SARS-CoV and on SARS-CoV-2^14^. It was indeed observed that most individuals infected with SARS-CoV-2 develop neutralising antibodies. The most potent ones are typically targeted against the S protein, particularly towards the RBD, which tends to be the most immunodominant region^15^. However, compelling evidence shows that SARS-CoV-2 genome has undergone evolutionary changes and diversification. Such an evolutionary pressure has caused most genetic mutations to be localised in the sequence of the S protein^16^. It has been demonstrated that these mutations mainly enhance the affinity of the spike RBD against the ACE2 receptor. Affected residues are mainly localised in the RBD, which leads to the impairment of viral neutralisation by Immunoglobulins (Ig) G and is responsible for immunological escape^17^. Differently from S protein, the N protein appears to be more evolutionarily conserved and less sensitive to selective immune pressure. Sequence homology and bioinformatic studies highlighted that N protein sequences display elevated homology among various SARS-CoV-2 variants that have emerged during the pandemic spread, and moreover, some immunodominant regions appear to be highly conserved^18,19^. This may facilitate the development of potential effective tools with enduring efficacy protein. Furthermore, as the N protein has been less investigated compared to S, further studies are needed to clearly elucidate nucleocapsid functions and its related immune response.

Within this context, this work describes the construction of different antibody libraries using blood samples collected from patients infected by B.1 SARS-CoV-2 strain (hCoV-19/Italy/LOM-UniSR10/2021, GISAID Accession ID: EPI_ISL_2544194). Our aim is to establish a stable and durable source of antibodies targeting SARS-CoV-2 antigens. From these libraries, we selected a panel of single chain variable fragments (scFv) against the N protein using the Intracellular Antibody Capture Technology (IACT)^20,21^.

Some scFvs were purified as recombinant proteins and displayed affinity for recombinant N in the nanomolar range. Other scFvs exhibited high intracellular binding activity to N protein when expressed in mammalian cells as intrabodies. Intrabodies are recombinant antibody domains expressed in mammalian cells by gene transfer, used as intracellular antibodies for phenotypic knockout or imaging^22^. Indeed, intrabodies represent a class of intracellularly stable molecules able to bind and target intracellular proteins in mammalian cells, to study their function by imaging, retargeting, interference, targeted degradation or direct neutralization strategies. Using intrabodies offers several advantages such as binding intracellular targets in different subcellular compartments. The binding domain of intrabodies can be equipped with a number of different effector domains, allowing, for instance imaging, degradation, cell death, to provide a diverse range of applications^23^. Altogether, this panel of recombinant antibody domains against SARS-CoV-2 N protein represents a novel platform for different research purposes and potential diagnostic applications.

## Results

### Human scFv libraries from COVID-19 recovered patients: construction and sequencing

**Figure 1:**
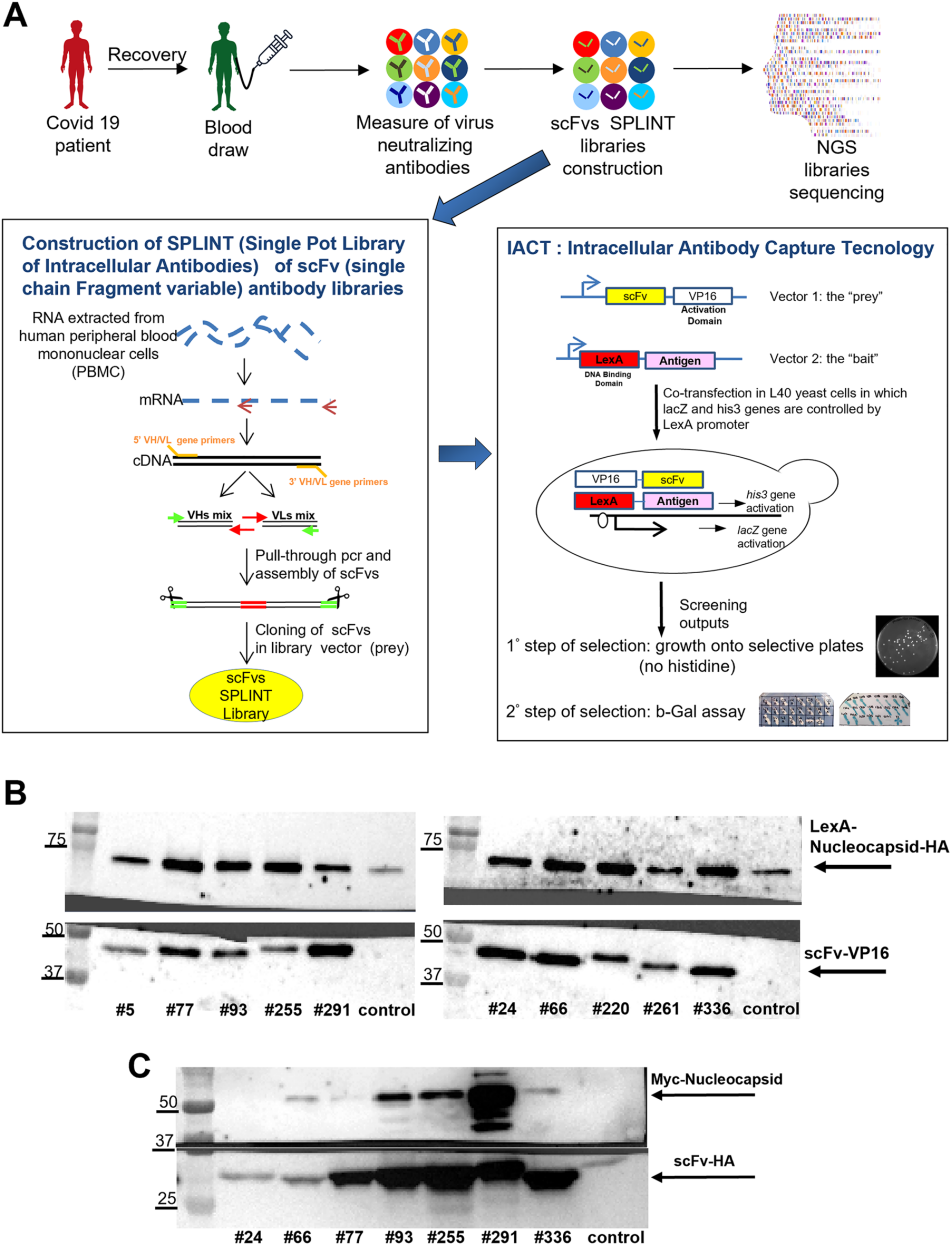
Schematic representation of scFvs library construction and IACT screening (**A**). Co-immunoprecipitation of Nucleocapsid full length protein with scFv anti Nucleocapsid in yeast (**B**) and in HEK 293 T cells (**C**). (**A**) Blood from patients recovered from Covid-19 was collected and tested for the presence of neutralizing antibodies. scFvs libraries were constructed from the RNA isolated from their peripheral blood mononuclear cells and sequenced. On the left panel, the schematic representation of the Single Pot Library of Intracellular Antibodies (SPLINT) library construction is shown. SPLINT libraries are then used in the Intracellular Antibody Capture Technology (IACT), schematically shown on the right panel. The bait and prey plasmids are co-expressed in yeast cells. In those cells in which bait and prey interact, his3 and lacZ genes are activated and these events allow selection. (**B**) Extracts from yeast strains expressing both the LexA-N-FL-HA protein (with LexA at the N-terminus and HA tag at the C-terminus) (69 KDa) and each of the anti N scFvs-VP16 (with VP16 at the C terminus) (#5, 77, 93, 255, 291, 24, 66, 220, 261, 336) or an unrelated scFv (#58F, anti AcK9H358^60^) (control) have been immunoprecipitated with anti HA antibody (top panels). Co-immunoprecipitated scFvs (~ 38.5 KDa) were detected with the anti VP16 antibody (bottom panels). (**C**) HEK 293 T cell extracts from cells expressing both the Myc-N-FL protein (with the Myc tag at the N-terminus) (46.8 KDa) and the individual scFvs-HA (with the HA tag at the C-terminus) (# 24, 66, 77, 93, 255, 291, 336 or an unrelated scFv (#58F, anti AcK9H358) used as negative control) have been immunoprecipitated with anti HA antibody (~ 28 KDa) (bottom panel). The co-immunoprecipitated Nucleocapsid protein was detected by the anti Myc antibody (top panel). Uncropped blots are presented in the Supplementary material section “Images of the original blots”.

Libraries of the immunoglobulin variable heavy (VH) and variable light chain (VL) of the IgM and IgG/IgA genes were generated from peripheral blood mononuclear cells (PBMC) of patients that had recovered from SARS-CoV-2 infection. Plasma samples were analysed to determine the titre of SARS-CoV-2 specific IgM, IgA, IgG neutralizing antibodies. PBMCs were then isolated from the blood sample of the 6 patients exhibiting the highest titre of neutralizing antibodies (see Suppl. Table 1) which serves as an indicative marker of robust, protective humoral anti-viral response. A detailed description of SPLINT (Single Pot LIbrary of INTracellular antibodies)^24^ libraries construction is reported in both Materials and Methods (M&M) and Supplementary material sections. The RNA extracted from PBMCs was retro-transcribed in cDNA using specific primer either for the IgM or the IgG/IgA repertoires (Fig. 1A). Thus, six cDNA libraries were prepared from the IgM and six from the IgG/IgA gene repertoires and transformed into *E.Coli* cells (M&M). Library complexity was estimated based on the total number of transformants obtained from colony forming unit (CFU) count (Table 1) (confidence interval (CI) for IgM libraries = 1,15 × 10^6^–1.05 × 10^7^ and CI for IgG/IgA libraries = 3.12 × 10^6^–7.87 × 10^6^). These estimates represent a lower limit to the actual library complexity, since they do not account for the possibility of more than one plasmid entering each colony-forming bacterial cell^25–28^.

**Table 1:** Library complexity determined as the number of total transformants obtained through CFU count.

Library #	IgM library transformation efficiency CFU	IgG/IgA library transformation efficiency CFU
1	1.68 × 10^7^	6.01 × 10^6^
2	6.98 × 10^6^	1.00 × 10^7^
3	5.80 × 10^6^	6.62 × 10^6^
4	1.87 × 10^6^	5.96 × 10^6^
5	2.57 × 10^6^	2.62 × 10^6^
6	1.00 × 10^6^	1.78 × 10^6^

The IgM and the IgG/IgA cDNA repertoires were reassembled as single-chain Fv (scFv) antibody domains, in the format of a yeast-two hybrid prey, and transformed into yeast cells forming the SPLINT library, for the selection of scFv antibody domains selectively and specifically recognizing the antigen bait^24^ (Fig. 1A).

The quality of the patient-derived libraries was assessed by Next Generation Sequencing (NGS). The VH and VL variable regions of the 12 scFv libraries were sequenced as described in the M&M section. To facilitate clonality assessment and to unambiguously estimate and assign distinct clonotypes, we obtained separate sequences for the VH and the VL variable regions through molecular barcoding with Unique Molecular Identifiers (UMI) (see M&M). Sequencing data demonstrated libraries’ quality, as testified by the large percentage of full-length sequences (80%) and hence the low number of sequences with stop codons (less than 20%). The combinatorial complexity, obtained by multiplying the diversity of VH by the diversity of VL, was consistently greater than 10^9^ (Suppl. Table S2–3). We compared the occurrence of clonotypes (as identified by the amino acid sequence of the CDR3 of either the VH and VL) of the 6 IgM libraries with those from a previously-described naive human IgM library of scFv^29^, and found very little overlap (Suppl. Fig S1).

This might be explained by the general expectation that the antibodies normally present after an immune response might normally be much skewed and enriched towards the target under investigation. Our data provide a direct sequencing demonstration of this prediction. More specifically, the little overlap observed between clonotypes from naive and immune libraries might reflect the fact that i) the naive IgM library was generated from the pooled PBMCs obtained from 4 subjects and might therefore contain a higher number of different clonotypes than a library from a single individual, such as our COVID-19 patient libraries and that ii) the libraries obtained from each individual COVID-19 recovered patient might be biologically skewed, displaying a polarisation of the immune response towards SARS-CoV-2 targets. Indeed, the occurrence of clonotypes among the different individual patients shows a good overlap (Suppl. Fig S2).

### IACT screening to isolate scFvs anti-N protein of SARS-CoV-2

We used the yeast two hybrid based IACT^20,21^ system to select scFvs against the SARS-CoV-2 N protein from the COVID-19 patients SPLINT libraries described above (Fig. 1A). The Dimerization Domain (DD) of SARS-CoV-2 N protein (aa 258–363) was chosen as a bait for the selection, as it is a functionally important and highly conserved domain across different Coronavirus strains as well as several SARS-CoV-2 variants of concern (Suppl. Fig S3-4). In addition, linear B cell epitope prediction (http://tools.iedb.org/bcell/, [PMID: 28472356]) showed that this domain has exposed epitopes and is highly immunogenic, which is consistent with other studies^30–32^. The IACT screening exploits L40 yeast strain co-transformed with antigen-bait/antibody-prey pairs. The antigen-bait is fused to a DNA binding domain (LexA) to be challenged with a library of recombinant antibody domains (scFvs) fused to the VP16 activation domain (the prey). A positive interaction between a prey and the bait activates transcription of the *his*3 gene, allowing survival on selective media (SD-WHL), as well as the* LacZ* gene as a second marker of interaction (Fig. 1A). The yeast strain expressing the LexA-DD of SARS-CoV-2 N protein was challenged with two of the IgG/IgA SPLINT libraries, obtained from the two patients with the higher neutralising antibody titer, in order to increase the diversity of the selectable scFvs. Out of the 360 initially-picked clones in the primary selection round, 34 clones resulted positive in the secondary screening against LexA-DD N protein, while remained negative when tested against the unrelated bait LexA-Synuclein. These clones were further tested against the full-length LexA-N bait encompassing residues 1–419 of SARS-CoV-2 N protein. Ten of these scFv domains (scFv # 5, 24, 66, 77, 93, 220, 255, 261, 291, 336) showed a strong interaction even after this challenge, as indicated by the yeast two-hybrid growth and survival assay in Suppl. Fig S5. The biochemical interaction of these scFv proteins with the LexA-full length N bait was further demonstrated by a positive co-IP from yeast cell lysates (Fig. 1B). For input expression refer to Suppl. Fig S6.

Details on the amino acid sequences of the complementarity determining region 3 (CDR3) of the VH and VL of each of the selected scFvs are reported in Suppl. Table S4.

### Expression of scFvs anti nucleocapsid as intrabodies in mammalian HEK293T cells

To evaluate the capability of the selected scFvs to interact with the N protein in mammalian cells, we cloned the coding sequences of the anti-N protein scFvs (# 5, 24, 66, 77, 93, 255, 261 291, 336) into the pEF1α-IRES-ZsGreen1 mammalian expression plasmid and additionally included an HA tag at the C-terminus. The coding sequence of the full-length N protein was instead cloned in the pP2A-mCherry-N1 plasmid construct by further adding a Myc tag at its N-terminus. Individual scFv domains were co-transfected along with N protein in HEK293T cells. After 48 h post -transfection, protein extracts were prepared and processed for scFv immunoprecipitation using HA agarose beads. Co-immunoprecipitated N protein was detected with an anti-Myc antibody (Fig. 1C). 5 out of the 7 tested scFvs (#66, 93, 255, 291, 336) showed a positive intracellular interaction with the N protein in HEK293T cells, while the remaining scFv#5 and 261 could not be assessed properly due to their sub-optimal expression levels in this cell line as shown in Suppl. Fig S7.

## Expression and purification of scFvs anti-N protein in *E. coli*

To investigate the in vitro interaction between scFvs and recombinant N protein, anti N scFvs # 5, 24, 77, 93, 255, 261, 291, 336 were expressed in *E. coli* and purified. scFvs # 66 was not expressed by the host. The detailed results of each purification are reported in Supplementary Materials. The scFvs produced in *E. coli* exhibited different yields (Table 2), as it is commonly observed for different scFv domains. Moreover, since scFvs are naturally prone to aggregation by intermolecular cross-pairing of their VH and VL variable domains^33–35^, size exclusion chromatography (SEC) for each scFv was carried out in order to obtain monomeric preparations (Suppl. Fig S8). As resulted by SEC analysis, scFvs #24 and #336 exhibited a good yield and purity and were obtained in the monomeric form, while scFvs #5, 93, 255, and 291 have a high tendency to oligomerize, as is often the case for many scFv fragments.

**Table 2:** Yield and aggregation propensity of scFvs expressed in E. coli.

scFv (pI)	Yield pre-SEC (mg/L)	Monomeric form obtained by SEC	Notes
#5 (8.48)	0.24 mg/L	NO	High tendency to form oligomers
#24 (9.21)	10.79 mg/L	Yes	
#77 (7.72)	0.04 mg/L	NA	Low yield
#93 (6.22)	13.06 mg/L	NO	High tendency to form oligomers
#255 (6.70)	1.175 mg/L	NO	High tendency to form oligomers
#261 (8.58)	0.045 mg/L	NA	Protein precipitation after refolding
#291 (8.50)	0.142 mg/L	NO	High tendency to form oligomers
#336 (7.72)	2.44 mg/L	Yes	

SEC: size exclusion chromatography; yield preSEC: yield of total scFv protein, before SEC.

### Characterization of the binding activity of anti-N scFvs to recombinant N protein

**Figure 2:**
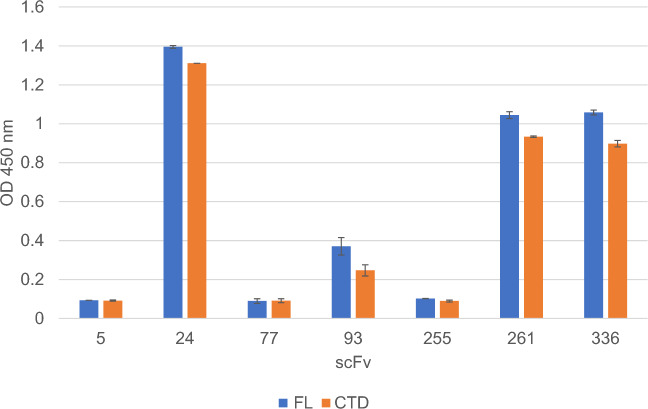
ELISA assay. The scFv #5, 24, 77, 93, 255, 261, 336 were tested for binding to the N-FL (blue) and the N-CTD (orange) protein, coated on the plate. The error bars represent the standard deviations. The assay was repeated twice.

A preliminary characterization of the binding properties of ion exchange (IEX)- purified scFvs for the N protein was carried out by Dot Blot (Suppl. Fig S9-10) and Enzyme-Linked Immunosorbent Assay (ELISA). Particularly, the ELISA was carried out by coating either the full-length (N-FL) or the C-terminal domain (N-CTD) of the N protein. This provides an indication about the binding strength between the scFvs and recombinant N protein in the two different formats. As shown in Fig. 2, scFv #24, 261 and 336 displayed a strong binding signal, with the scFv #24 resulting as the best binder; scFv #93 exhibited an intermediate signal, while scFvs #5, 77 and 255 exhibited a weak binding both to N-FL and N-CTD protein. scFv proteins showed no binding preference for the recombinant C-terminal domain (N-CTD) or the full length N protein. As expected, signals from the unrelated scFv, the no-coating and no-primary samples were equivalent to those from the blank ones. Despite its strong binding to recombinant N protein, scFv #261 was not further characterized and its analysis discontinued, due to its poor purification yield (Table 2).

**Figure 3:**
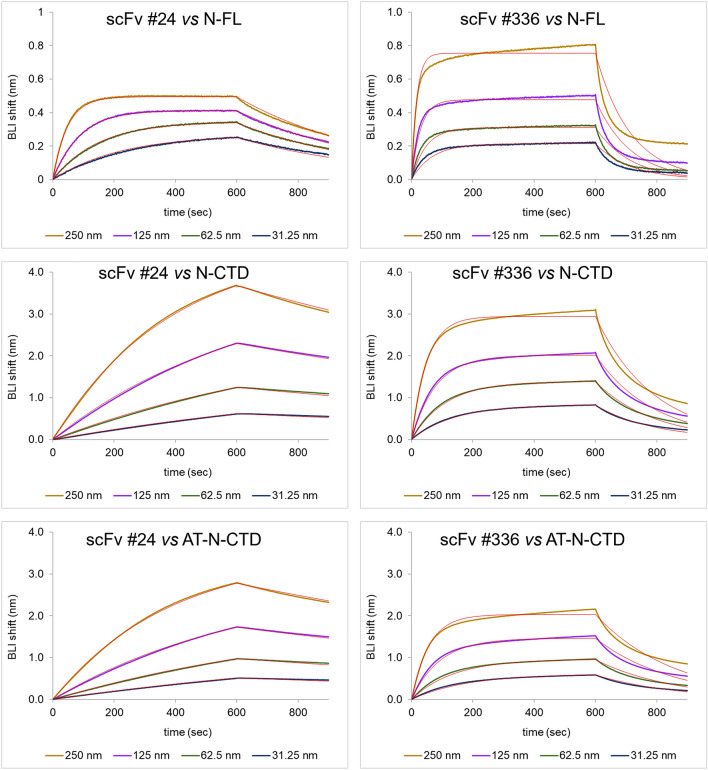
BLI binding analysis of scFvs to immobilized recombinant N-proteins constructs. Each panel represents sensorgrams of BLI experiments performed immobilizing the recombinant N-protein construct to SA BLI sensors. Red lines represent the fitting curve for each tested concentration.

We then quantified the kinetic parameters for the binding between the recombinant N protein and scFvs #24 and #336 (which have favourable properties of expression levels and monomeric status) using a Biolayer interferometry (BLI)-based binding assay. Biotinylated N protein was loaded on BLI Streptavidin (SA) biosensor surface and the interaction with the scFv analytes was probed. Both scFv #24 and #336 resulted to have an equilibrium dissociation constant (K_D_) for N-protein in the nanomolar range, with #24 showing slower dissociation (i.e. longer residence time) and higher binding affinity (lower K_D_) (Table 3, Fig. 3). N-FL showed a higher affinity for scFv #24 with respect to the N-CTD form, primarily due to its faster association rate (k_a_), and its higher dissociation rate (k_d_). This indicates that also the N-terminal region of the N protein may be involved in binding. The biotin-labelling at the only N-terminal amine for the AT-N-CTD (AviTag-N-CTD) construct did not result in a significant improvement in binding affinity, suggesting that lysine residues in the N-CTD are very likely not involved in the interaction with the scFv #24. ScFv #336 sample showed reduced homogeneity with some trace of oligomeric forms, resulting in fast dissociation of the analyte, which was more pronounced for binding with the N-FL.

**Table 3:** Binding parameters for the interaction between the recombinant N protein and scFvs #24 and #336.

Analyte	Ligand	KD(nM)	KD Error (nM)	ka(1/Ms)	ka Error	kd (1/s)	kd Error	Full R^2^
scFv #24	N-FL	27.8	0.0	7.64E + 04	1.03E + 0.2	2.13E-0.3	2.04E-0.6	0.9976
	N-CTD	74.9	0.2	8.35E + 03	1.71E + 0.1	6.25E-0.4	1.30E-0.6	0.9994
	AT-N-CTD	66.3	0.2	8.27E + 0.3	1.70E + 0.1	5.48E-0.4	1.28E-0.6	0.9994
scFv #336	N-FL	196	1.6	6.42E + 0.4	4.53E + 0.2	1.26E-0.2	5.28E-0.5	0.9557
	N-CTD	95.1	0.4	5.74E + 0.4	2.10E + 0.2	5.46E-0.3	5.28E-0.5	0.9944
	AT-N-CTD	69.4	0.3	5.58E + 0.4	2.02E + 0.2	3.88E-0.3	6.49E-0.6	0.9924

k_a_ and k_d_ values were estimated by globally fitting the BLI response intensity (nm) as a function of scFv concentration (nM) with the Octet Data Analysis Software, using a 1:1 Langmuir binding model. K_D_ was calculated as k_d_/k_a_. Errors for ka and kd are reported as standard errors of the mean calculated from the fitting curves.

### Evaluating the intracellular interaction of anti-N scFvs with Nucleocapsid

The antiviral activity of anti-nucleocapsid antibodies do not seem to stem primarily from conventional neutralizing activity, but rather from alternative mechanisms such as NK-mediated cytotoxicity against infected cells^36^ and their ability to bind both cellular and freely circulating Nucleocapsid (reviewed in Focosi et al.^37^). It was therefore not unexpected to find that the recombinant antibodies we developed lacked neutralizing activity, as shown in Suppl. Fig S11.

Given our observation of antibody binding to extracellular nucleocapsid protein and previous reports suggesting its contribution to pathogenicity when exposed on the cell surface or secreted^37^, we further explored the potential of anti-N scFvs to interact intracellularly with the natural virus N protein in the in-vitro model of SARS-CoV-2 using a co-immunoprecipitation assay.

**Figure 4:**
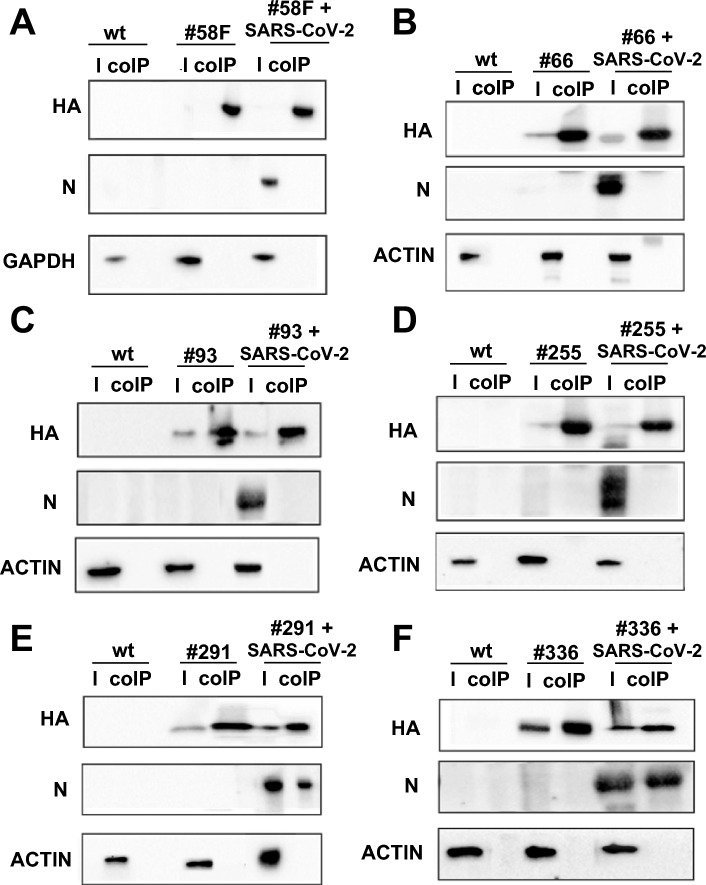
Co-immunoprecipitation of SARS-CoV-2 viral N protein with anti-N scFvs expressed in Vero-TMPRSS2 cells. Every panel displays three types of samples: (i) control cells not transfected and not infected (wt), ii) cells transfected with the scFv and (iii) cells both transfected with the scFv and infected with SARS-CoV-2 B.1 strain. For each sample the input for total cell lysate (I) and the co-immunoprecipitated proteins (co-IP) were loaded. (**A**): Co-immunoprecipitation of unrelated scFv (#58F) used as negative control. (**B**): Co-immunoprecipitation of scFv#66. (**C**): Co-immunoprecipitation of scFv#93. (**D**): Co-immunoprecipitation of scFv#255. (**E**): Co-immunoprecipitation of scFv#291. (**F**): Co-immunoprecipitation of scFv#336. Immunoprecipitated scFvs were detected using anti-HA antibody and co-immunoprecipitated N protein was identified using an anti-N antibody. Actin was used as loading control. Uncropped blots are presented in the Supplementary material section “Images of the original blots”.

Protein extracts from Vero-TMPRSS2 cells, first transfected with the selected scFvs (cloned in the pEF1α-IRES-ZsGreen1 plasmid) and then infected with the SARS-CoV-2 B.1 strain, were processed for immunoprecipitation with an anti-HA antibody. As shown in Fig. 4 the viral N protein was effectively co-immunoprecipitated by the intracellularly expressed scFv#291 and scFv #336 intrabodies, demonstrating that these scFv intrabodies productively interact in the cell with the intracellular N protein, also when it is virally encoded and not only when it is co-expressed by plasmid transfection. This effective intracellular interaction, however, does not determine an inhibition of viral titer in the cell supernatant.

## Discussion

In May 2023, more than 3 years after COVID-19 was classified as a pandemic, the World Health Organization (WHO) declared the end of COVID-19 as a global health emergency, with 765 million of cases and 6.9 million of deaths reported worldwide, and more than 5 billion vaccinated people mainly in the wealthiest countries^38,39^.

However, COVID-19 continues to take a heavy toll on personal health, healthcare systems, and economies around the globe. In the early years of the pandemic, attention focused primarily on the SARS-CoV2 spike protein due to its role in the viral entry into the cells and elicitation of neutralizing antibodies^11^. More recently, the research has focused on the N protein because of its importance in diagnosis^11,40,41^, for the development of new therapeutics^11,42,43^ and as an antigen in the second generation of vaccines^41,44^. As described above, the N protein of SARS-CoV-2 plays a crucial role in viral replication, genome packaging, and evasion of the immune system^10–12^. In contrast to the genetic variability observed in S, the N protein remains more conserved^18,19^ and is highly immunogenic, inducing not only an antibody response, but also a strong T-cell response^45,46^.

In this study we have established a stable and durable source of antibodies against SARS-CoV-2 by generating 12 recombinant single-chain fragment variable (scFv) libraries that exploited the immune response of COVID-19-covalescent patients. We estimated library complexity as the number of total transformants obtained through CFU count (Table 1). These estimates represent a lower limit to the actual library complexity. Indeed, it is commonly believed that *E. coli* rarely takes up more than one plasmid per cell upon transformation. Nevertheless, as Hanahan^25^ showed in his classic paper, cotrasformation of *E. coli* can be very high, since “About 1% of the cells became transformed, and 70 to 90% of these, were doubly transformed at equimolar ratios”. A more recent study^26^ re-examined the question of plasmid copy number upon co-transformation with plasmids with the same, or with different, origins of replication and concluded that multiple plasmids are able to stably persist in bacteria for several growth cycles and that “plasmid persistence is far more common than generally realized”. In practical terms, the demonstrated plasmid persistence had been exploited to generate increased diversity in phage display antibody libraries^47^, as in our case in this manuscript. A very recent study^27^ also shows multiple plasmid persistence using plasmids that direct the expression of fluorescent proteins. In a recent paper, Goldsmith et al.^28^examined carefully the issue of double transformation and how to control it, and concluded that the double transformation events can be quite frequent. All in all, we conclude therefore that these events could contribute to the antibody library diversity, and could be taken into account to estimate library diversity prior to selection procedure.

From these libraries, we selected a panel of scFvs against the SARS-CoV-2 N protein, by means of the IACT.

We focused our attention on 10 scFvs that showed the strongest interaction in the yeast two hybrid assay, by analysing them both as intrabodies and as purified recombinant proteins. Our aim was to identify promising candidates (i) as intrabodies in order to proceed in further research on the intracellular infection mechanism of the virus, (ii) as purified scFvs to be used to set up new diagnostic tools, in the same format or reformatted to whole Ig.

For use as intrabodies, we identified scFvs #291 and #336 as the best candidates, as they show strong interaction with the full-length recombinant N protein in both yeast and mammalian cells, and for their ability to co-immunoprecipitate the viral N protein in a cellular model of SARS-CoV2 infection.

Indeed, the intrabodies scFvs #291 and #336 were used in Vero-TMPRSS2 cells infected with SARS-Cov-2 virus. Despite not hindering virus replication, scFv #291 and #336 demonstrated the capability to bind intracellularly the viral N protein. This interaction suggests a potential contribution to the broader spectrum of antiviral mechanisms outlined elsewhere and extending beyond mere neutralizing activity^37^. These scFvs could be exploited as tools to investigate the complex virus biology in in vitro models, i.e. targeting the scFvs to different cell compartments by specific consensus signals for studying their effect on virus life cycle. The anti N scFv fragments might be equipped with a range of different effector domains (such as for example with fluorophores, degron domain) to investigate the cellular functions of N protein in viral biology. Concerning this point, future studies could investigate the possibility of adding a degron domain to the intracellularly expressed scFvs in order to escort the intercepted viral N protein to intracellular degradation and thus inhibit viral replication^48,49^.

The intrabody-mediated targeting of the N protein to degradation might be imagined/designed as a future therapeutic tool. Ideally, these drugs can be administered intranasally (as mRNA or directly as proteins) through their encapsulation in liquid nanoparticles.

On the other hand, the scFvs expressed and purified as recombinant proteins in *E. coli* were extensively analysed to determine their expression and binding properties. Interestingly, this analysis led to the identification of scFv #24 and #336, which are characterised by high yield and purity as well as K_D_ in the nanomolar range for N protein, suggesting their potential use as diagnostic tools.

Indeed, the need for efficient diagnostic tools remains urgent. Most current techniques for the detection of SARS-CoV2 infection^40^ are based on the detection of Nucleocapsid or anti-N antibodies^11^, and rely on sensitive and sophisticated techniques. However, these methods have the limitation of not being readly manageable as point-of care methods. As a consequence, there are ongoing efforts to develop simpler, low-cost, highly sensitive and specific point-of-care methods, possibly based on easily collected biological fluids such as saliva. Methods based on biosensors could be a good prospect for future point-of-care diagnostics and our scFvs could be used to develop new tools displaying these features^11,40,50^, exploiting both their small dimension and recombinant nature that allows them to be engineered in versatile ways.

It is interesting to note that scFvs working better as intrabodies do not always correspond to those working better as purified protein. This is not surprising and can be explained by the differences between the cytosolic environment (given its peculiar ionic strength, reducing environment, etc.) and the buffer used for in vitro purification. In this respect, the analysis of scFvs carried out in both configurations performed in this work allows the selection of the most interesting candidates depending on the application to be performed. In this respect, the scFv #336 represented an exception, showing high binding properties to Nucleocapsid protein both in cells and in vitro systems.

In summary, in this study we generated fully sequenced libraries of human antibodies against SARS-CoV-2, which provide an immortalized and extensive resource for the isolation of human antibodies against SARS-CoV-2 antigens, as well as, in the future, for the isolation of human antibodies against other proteins of interest. From these libraries, we report the selection and characterization of a panel of anti N scFvs with remarkable properties that can be used for research and diagnostic purposes.

## Materials and methods

### Enrolment of patients who have recovered from COVID-19, collection of biological samples and isolation of PBMC

The blood samples of 40 patients who had recovered from COVID-19 (SARS-CoV-2 B.1 strain) were collected by the Virology Unit of the Azienda Ospedaliero Universitaria Pisana (AOUP) and screened for the presence of COVID-19 specific neutralizing antibodies with a titre 1/40 (11 patients), 1/80 (10 patients), 1/160 (15 patients), 1/320 (3 patient) and 1/640 (1 patient) (Suppl. Table S1). Two sera were further specifically tested by ELISA in different dilutions (1:2, 1:10, 1:100), to confirm the presence of anti-N antibodies. Donors provided their written informed consent of the protocol approved by the Ethics Committee of Area Vasta Nord-Ovest Toscana (CEAVNO Prot.n° 17,968). 12 libraries were derived from blood samples of patients with a neutralizing antibody titer > 1/160, of which the RNA extracted from PBMC was of good quality and quantity to proceed with libraries construction (see Suppl. Table S1). PBMC were isolated by density gradient following Lymphoprep protocol (Stemcell Technologies, # 07801) and then 10^7^ aliquots were frozen in Trizol (Thermo Fisher Scientific, # 15596026) at − 80 °C until use.

### RNA extraction and IgM, IgG and IgA cDNA library preparation

Total RNA was isolated by acid guanidinium thiocyanate-phenol chloroform extraction (using TRIzol RNA Isolation Reagents, Thermo Fisher Scientific, #15596026) from the PBMC of the 6 COVID-19 patients with the highest neutralising antibodies titer. RNA integrity was assayed by agarose gel electrophoresis. The total RNA (5 µg) was first subjected to DNase treatment using the DNAse I Amplification grade kit (Merck, AMPD1-1KT) and then retro-transcribed in cDNA using specific primer either for the IgM or the IgG/IgA repertoires. Specifically, the primers used for the IgM repertoire were: HuIgMFOR: 5′ TGGAAGAGGCACGTTCTTTTCTTT 3′; HuCκFOR: 5′ AGACTCTCCCCTGTTGAAGCTCTT 3′; HuCλFOR: 5′ TGAAGATTCTGTAGGGGCCACTGTCTT 3′, while the primers used to generate the IgG/IgA specific cDNA were: HuIgG1–4CH1FOR: 5′ GTCCACCTTGGTGTTGCTGGGCTT 3′; HuIGA_r1: 5’ GCGACGACCACGTTCCCATCT 3′; HuCκFOR: 5′ AGACTCTCCCCTGTTGAAGCTCTT 3′; HuCλFOR: 5’ TGAAGATTCTGTAGGGGCCACTGTCTT 3′.

### Construction of human SPLINT libraries for IACT selection

The IgM and IgG/IgA cDNA libraries from each patient were first reformatted to scFv fragments, which were then used to prepare SPLINT libraries in the scFv format, in the pLinker220 SPLINT vector^24^. scFvs antibody libraries were assembled from the IgM cDNA and from the IgG/IgA cDNA following a protocol modified from Marks and Bradbury^51^ and detailed in Fantini et al*.*^29^. Briefly, IgM or IgG/IgA cDNA was used as a template to amplify VH and VL regions. Primers were designed to anneal to the external framework regions of the V genes. All the VH and VL subclasses were amplified together in a single reaction (one reaction for the VH, one for the light Vλ and one for the Vκ) using a mix of the 5’ and 3’ primers available (see Supplementary material). Construction of each library occurred in five steps as shown in Suppl. Fig. S12: (i) amplification of VHs, Vκs and Vλs from Ig cDNA; ii) construction of a linker (G4S)3 with primers specific for VHs, Vκs and Vλs; iii) assembly of each variable class (VHs, Vκs and Vλs) with the specific (G4S)3 linker (VH, Vκ and Vλ blocks); iv) pull through PCR of VH block with Vκ blocks and VH blocks with Vλ blocks to generate scFv objects, and addition of restriction sites for BssHII at 5’ and for NheI at the 3’ to the scFv pull through products; v) ligations of BssHII/NheI digested pull through products to pLinker220 SPLINT vector. Each step is well detailed in Supplementary material (Suppl. Table S5-S12). Ligation products of either the IgM or the IgG/A scFv library were transformed by electroporation in E-Max Efficiency DH5α (as described in Supplementary material) and grown in LB with Sea Prep Agar^52^, in the presence of ampicillin. LB + Sea Prep Agar is a semi-solid medium in which bacteria are entrapped and grow in a tridimensional mesh. This is important to preserve the maximum diversity of all the plasmids composing the library. Indeed, growth in such semi-solid media allows propagation of a larger number of clones compared to growth in the liquid medium alone, in which the stronger clones could, and will, take over.

An aliquot of the inoculum was plated on LB-Agar + ampicillin plates with serial dilutions to evaluate the transformation efficiency. Assuming that each transformed bacterium takes one copy of each plasmid DNA composing the library, we can define the lower bound of the library complexity as the number of total transformants obtained, as determined by colony forming units (CFU) count.

The 6 IgM-derived and 6 IgG/IgA-derived scFv SPLINT libraries were stored, as plasmid DNA, at − 20 °C and used for IACT selections against the N nucleocapsid protein.

### NGS sequencing of the scFv libraries

Each of the 12 SPLINT libraries (6 for the IgM and 6 for the IaG/IgA repertoire) were sequenced by the ION TORRENT technology by Genomnia. For each scFv library, indipendent VH and VL libraries were  constructed as follows. VH and VL were amplified using primers (VH_fus_F- VH_fus_R and VL_fus_F–VL_fus_R_BXX) displaying at their 3′ end the region of the pLinker220 plasmid flanking the VH or VL. Ion Torrent adaptors were present at the 5′ end. In this step, Unique Molecular Identifiers (UMI) consisting of 13 degenerate bases are also introduced in the reverse primer used to amplify the VH and VL regions. The amplified VH and VL were then subjected to a second PCR using the primers A_fus–trP1_fus to build the fusion library (primers are listed in Suppl. Table S13 and S14). Sequencing was performed on the Ion Torrent platform IonS5 displaying a 400 bp chemistry on a 530 chip. Both types of sequencing libraries were constructed by Genomnia. Each VH library was individually sequenced to a 400 bp read on a 530 chip, yielding approximately 20–25 million reads per sample. The VL libraries, on the other hand, were barcoded and sequenced in a pool of three libraries from different patients, aiming to obtain 6–10 million reads per library. All sequencing runs were of good quality, with an average of 73% of the reads mapping to immunoglobulin genes used for clonotype identification (i.e. less than 30% polyclonal reads) and less than 20% low-quality sequences. Combinatorial complexity, obtained by multiplying the number of different VH by the number of different VL sequences, was consistently greater than 10^9^ (Suppl.Table S2 and S3), and the presence of sequences with stop codons was < 20%.

### Bioinformatics analysis of library sequencing and Immuno-fingerprinting analysis protocol

The bioinformatics analysis of the libraries was based on the UMI molecular barcodes introduced in the first two amplification cycles of the VH and VL regions. Amplification and sequencing errors are corrected by grouping reads based on UMI^53^.

The analytical pipeline developed involved the use of the MIGEC^54^ + MiXCR^55^ softwares for assembly on CDR3, without clustering, and the VDJtools^56^ software to generate statistics and graphs starting from the clonotypes assembled by MiXCR. This software assembles identical and homologous reads into clonotypes, correcting for PCR and sequencing errors and identifies as a clonotype a group of nucleotide sequences having the same CDR3 and the same variable (V) genes.

### Preparation of the bait strain for IACT

The DNA sequence encoding the dimerization domain (DD) of SARS-CoV-2 N protein (aa 258–363) or the full-length protein (aa 1–419), amplified from the Addgene plasmid#141391, was cloned in pMicBD1 plasmid in frame with the DNA binding domain LexA (yielding the plasmid pMicBD1-LexA-DD N Protein or pMicBD1-LexA N Protein), and transformed in the L40 yeast strain. Cells were grown in 1% Yeast Extract, 2% Bacto Peptone, 2% Glucose, at pH 5.8 to an OD at 600 nm of 0.6. Subsequently, they were washed in 1xTE (10 mM Tris, 1 mM EDTA, pH 7.5), and resuspended in 0.5 ml of 1xTE/1xLiAC (10 mM Tris, 1 mM EDTA, 0.1 M Lithium acetate dehydrate pH 7.5). Yeast cells (100 µl) were then added to 100 µg of salmon tested DNA (STD) and 200 ng of pMicBD1-DD N protein plasmid with 600 µl of 50% PEG/1xTE/1xLiAC (40% (w/v) PEG 4000, 10 mM Tris–HCl, 1 mM EDTA, 0.1 M lithium acetate dehydrate pH 7.5) and shaken at 150 rpm for 30 min at 30 °C. After adding DMSO (70 µl), cells were heat-shocked at 42 °C for 15 min, placed on ice for 2 min, centrifuged, resuspended in 100 µl of 1xTE and plated on Synthetic Designed liquid minimal medium lacking tryptophan (SD-W) plates.

The DNA sequence encoding the full-length protein (aa 1–419) of SARS-CoV-2 N protein was also amplified from the Addgene plasmid#141391 and subsequently cloned in the pMicBD1 plasmid in frame with the LexA DNA-binding domain. The resulting final construct, named pMicBD1-LexA N Protein, was then transformed in the L40 yeast to be used in secondary screening.

### scFvs anti N selection using IACT

For IACT screening^26,27^, the L40 yeast strain was transformed with pMicBD1-LexA-DD N Protein plasmid to express the LexA-DD N protein bait. Cells were grown O/N at 30 °C in SD-W media. The O/N culture was then diluted in 1 l of pre-warmed rich medium YPAD (1% Yeast Extract, 2% Bacto Peptone, 0.01% Adenine, 2% Glucose, pH 5.8), and cultured from an OD at 600 nm of 0.3 to 0.6. Cells were centrifuged, washed in 150 ml of 1xTE, and resuspended in 15 ml of 1xTE/1xLiAC. Salmon testes DNA (STD) (10 mg) and a mix of two of the IgG/IgA SPLINT hscFv libraries (250 µg each) (derived from the two patients with the higher neutralizing antibody titer library #4 and #5), previously cloned in the pLinker220 prey plasmid, were added. For transformation into the L40 yeast strain expressing the LexA-DD N protein bait, the mixture of the two IgG/IgA scFv libraries was transferred in a flask with 140 ml of 50% PEG/1xTE/1xLiAC and incubated at 150 rpm for 30 min at 30 °C. Following DMSO addition (17.6 ml), cells were heat-shocked at 42 °C for 15 min under gentle mixing. After placing flasks on ice for 5 min, cells were washed three times with YPA (1% Yeast Extract, 2% Bacto Peptone, 0.01% Adenine, at pH 5.8), and recovered in 1 l of YPAD for 1 h at 30 °C. Cells were washed three times with SD-WHL (SD without Tryptophan, Histidine and Leucine), resuspended in 5 ml of SD-WHL and plated on SD-WHL Petri dishes. Following an incubation of 4–5 days at 30 °C, 360 clones were picked and re-streaked onto SD-WHL and SD-WL plates. A liquid β-galactosidase (β-gal) assay, adapted from Möckli & Auerbach^57^, was performed using a 96-well plate. Briefly, a small amount of the biomass from single colonies was resuspended in 50 µl lysis buffer (20 mM Tris HCL pH 7.5, 333 U/ml lyticase) and incubated for 2 h at 37 °C. 50 µl of a solution made of 60 mM Na_2_HPO_4_, 40 mM NaH_2_PO_4_, 10 mM KCl, 1 mM MgSO_4_, pH 7.0, X-gal at 20 mg/ml (170 µl), and β-mercaptoethanol (30 µl) were added to each well and incubated for 2 h at 37 °C. Strong prey–bait interactions were identified by the development of blue color. 70 clones resulted positive to the β-gal assay.

### Secondary screening

The 70 individual clones were subjected to secondary screening by transforming them into either the pMicBD1-DD N protein yeast strain or a yeast strain expressing an unrelated bait (pMicBD1-Synuclein) to exclude anti LexA positive scFvs, using the protocol described above in the “bait strain preparation” section. The 48 scFvs that resulted positive were then screened in a yeast strain expressing the full-length SARS-CoV-2 N protein (aa 1-419) (pMicBd1-N Protein) and 34 resulted positive for the interaction with the full-length protein.

### Co-immunoprecipitation of anti N protein scFvs and N Protein from L40 yeast co-expressing cells

The L40 yeast strains co-expressing LexA-N-HA protein (from the plasmid pMicBD1-LexA-N Protein) and either one of the 10 (# 5, 24, 66, 77, 93, 220, 255, 261, 291, 336) anti N protein scFvs-VP16 (from the pLinker220 plasmid), were grown O/N at 30 °C in SD-WL media. The O/N culture was diluted in 20 ml of pre-warmed rich medium YPD and cultured from an OD at 600 nm of  0.3 to 0.6. Cells were centrifuged and resuspended in 250 μl of 1xTE buffer (Tris–EDTA buffer) with protease inhibitor. Glass-beads (Merck, #G8772) were added to each sample covering about ¾ of the volume of the resuspended cell pellet. Lysis was performed with eight cycles of 1-min vortex at maximum speed followed by 1 min on ice. The cell extract was recovered, transferred to clean 1,5 ml Eppendorf tubes and Triton-100 was added to a final concentration of 2%. Each sample was vortexed at maximum speed for 30 s and centrifuged at 14,000 g for 15 min at 4 °C. The supernatant was recovered and protein concentration was determined by Bradford assay.

500 µg of protein extract were used for immunoprecipitation (IP) of the N protein. The anti HA agarose beads (Pierce, #26181) for IP were pre-incubated with BSA 10 µg in 250 µl of 1 × TBS, for 1 h at room temperature (RT). The blocking solution was removed and protein extract was incubated with anti HA agarose beads in rotation O/N at 4 °C. The next morning, beads were recovered and washed 6 times with 1X TBS + 0.05% Tween-20. The beads were resuspended in 30ul of 2X Laemmli buffer, boiled for 10 min and the eluates were analyzed by 13% SDS-PAGE, blotted onto nitrocellulose membranes, and blocked in 5% fat-free Milk in Tris-buffered saline (TBS) for 1 h at RT. The nitrocellulose membrane was then cut in half directly above the 50KDa protein marker. The upper part was incubated at 4 °C O/N with rabbit anti LexA (1:1000, Sigma # 06–719) to detect N protein immunoprecipitation, while the second half of the membrane was incubated at 4 °C O/N with rabbit anti-VP16 (1:1000, Sigma, #V4388) to detect co-immunoprecipitated anti-N scFvs. Blots were then washed 3 times in TBS-T (TBS + 0.1% Tween-20) for thirty minutes and the membranes incubated with secondary anti-Rabbit-HRP (1:2000, Santa Cruz Biotechnologies, #sc2004) for 1 h at RT. Following three washes in TBS-T for thirty minutes, membranes were incubated with Clarity Western ECL Substrate (Biorad, #1705061) and acquired through a Chemidoc XRS instrument.

### Co-immunoprecipitation of anti N scFVs and N Protein from HEK 293 T co-expressing cells

The coding sequence of the 9 anti N protein scFvs (# 5, 24, 66, 77, 93, 255, 261, 291, 336), displaying an HA tag at the C-terminus, were cloned in the mammalian expression plasmid pEF1α-IRES-ZsGreen1 (Clontech, # 631976). The scFv#220 was not cloned because its coding sequence harboured the same restriction site (BssHII) used for subcloning from the pLinker220 yeast vector to the pEF1α-IRES-ZsGreen1 vector. The coding sequence of the full-length N protein, together with a Myc tag at the N-terminus, was cloned in the pP2A-mCherry-N1 plasmid (Addgene, #84329). HEK 293 T cells were plated in 60 mm petri dish (500,000) and co-transfected on the following day with pP2A-mCherry-N protein-Myc and pEF1αZsGreen1-scFv-HA using the Effectene Transfection Reagent (Qiagen, # 301427). Briefly, 1 µg of each plasmid DNA was diluted in 150ul of buffer EC and 16 ul of Enhancer and the mixture was incubated at RT for 5 min. After this period, 50 μl of Effectene were added and the solution was mixed through 10-s vortexings and subsequently incubated at RT for 20 min to allow transfection complex formation. During incubation, cells were washed once with PBS and 4 ml of fresh growth medium (DMEM + 10% FBS penicillin/streptomicin 100 U/ml) were added. Following the 20-min time, 1 ml of fresh growth medium was used to dilute transfection complexes, which were immediately added drop-wise onto the plated cells. 48 h post transfection, cells were harvested and lysed in 120 µl of 1X PBS, 1% Triton-X100, 1% NP40 and protease inhibitor cocktail (Roche, #11873580001) for 1 h on ice using a syringe with 28-gauge needle for homogenization. The extract was then spun at 16000 g for 15 min. The supernatant was transferred to a new tube and protein concentration was determined by Bradford assay. 400 µg of protein extract were used for immunoprecipitation (IP) of the scFv using the same protocol described above. The nitrocellulose membrane was cut in two halves right above the 37 KDa protein marker. The upper part was incubated at 4 °C O/N with HRP-anti Myc (1:1000, Sigma, #16-213) to detect co-immunoprecipitated N protein, while the lower part of the membrane was incubated at 4 °C O/N with HRP anti-HA (1:1000, Roche, #12013819001) to detect scFv immunoprecipitation. Blots were then washed 3 times in TBS-T for thirty minutes and the membranes incubated with Clarity Western ECL Substrate (Biorad, #1705061) and acquired through a Chemidoc XRS instrument.

## *E. coli* expression and purification of scFvs anti-N protein

Anti-N protein scFvs were subcloned by restriction digestion with the BssHII/NheI enzymes into the pGIO1 vector^58^ in an upstream position with respect to the V5-Tag coding sequence, and subsequently transformed into *E. coli* BL21(DE3) pLysS (Novagen). Positive clones were grown at 37 °C in LB medium with kanamycin until a 600 nm absorbance value of 0.9 was reached. Cells were then induced by 1 mM IPTG (Isopropil-β-D-1-tiogalattopiranoside, Merck) for 4 h at 37 °C. All scFvs were produced in the form of insoluble aggregates. Isolation and resolubilization of inclusion bodies and refolding of recombinant proteins were performed following the same protocol for all scFvs. Briefly, induced cells were lysed to isolate inclusion bodies, which were in turn resolubilized in guanidine hydrochloride 6 M as explained in Rudolph et al*.*^59^. Resolubilized proteins were folded at 4 °C into the folding buffer (0.1 M Tris–HCl pH 8.5 (Applichem), 0.4 M L-Arginine (Applichem); 5 mM EDTA (Sigma), 375 μM L-Glutathione oxidized (Applichem)) by pulse renaturation, i.e., by adding protein at a final concentration of 35 ug/ml every hour, to a volume of refolding buffer calculated to allow a 30-fold dilution of the guanidine concentration in the protein sample. The folded scFvs were then dialyzed, centrifuged at 14,000 g for 40 min at 4 °C and purified by ion exchange (IEX) chromatography in an AKTA Purifier system, using different conditions depending on their isoelectric point.

Specifically, scFvs #5, #261, #291 were dialyzed against 20 mM sodium phosphate buffer pH 7, while scfv#77 against 20 mM sodium phosphate buffer pH 6.6. After dialysis, the scFvs were loaded onto a HiTrap SP column (1 or 5 ml) (GE Healthcare). Differently, the scFv #255 was dialyzed against 20 mM Tris HCl pH 8.8 buffer and loaded onto a HiTrap Q HP column 1 mL (GE healthcare). scFvs #24, #93, #336 were all dialyzed against 50 mM sodium phosphate buffer, at pH 7 for scFv #24, pH 7.8 for scFv #93 or pH 6.6 for scFv #336, and loaded onto a HiPrep SP Sepharose 16/10 (GE healthcare). For all the scFvs, after sample loading, the columns were washed with two column volumes (CV) of dialysis buffer and then the scFvs were eluted in 10 CV linear gradient from 0 mM to 1 M NaCl in the same buffer. In each step of the purification, the purity of the samples was checked by overloading an SDS-PAGE. Purification profiles of the scFvs can be found in Supplementary material (Suppl. Fig S13-S23).

### Size exclusion chromatography of the scFvs anti-N protein

Size-exclusion chromatography (SEC) on IEX-purified scFvs, was carried out to analyse the aggregation state of the samples. The scFvs were loaded onto Superdex 200 or 75 Increase 16/300 SEC columns (Cytiva) pre-equilibrated in Phosphate-buffered saline (PBS) pH 7.5, 0.5 mM EDTA. Proteins were eluted in the same buffer. The purity of the samples was checked by overloading an SDS-PAGE.

### Production of recombinant N protein

Three different constructs for N protein were used: N protein full length (N-FL, residues 1-419), N protein C-terminal dimerization domain (N-CTD, residues 247-364), and N-CTD with AviTag™ at the N-terminal (AT-N-CTD). The dsDNA sequence encoding for SARS‐CoV‐2 N-FL (NCBI reference entry NC_045512.2)^2^ was codon-optimized for *E. coli* expression, and purchased from GenScript. It was then inserted into an in-house derivative of the pET‐9d expression vector by restriction cloning with NcoI/NotI enzymes so that it encodes a hexa-histidine tag followed by a GST tag, a TEV protease cleavage site and the full-length N protein.

The plasmid encoding for N-CTD was obtained by two sequential deletion subclonings, the first aimed at the removal of the N-terminal region of N-FL (N-NTD, residues 1-246), and the second aimed at the removal of the C-terminal region encoding for residues 365-419. In the first step, the N-FL plasmid was used as a template, and amplified using a pair of primers annealing to the DNA regions flanking the N-NTD coding sequence. The resulting vector was then used as template for the second step, and amplified using a different pair of primers annealing to the DNA regions flanking the coding sequence for residues 365-419. For AT-N-CTD, the N-CTD plasmid was modified by inserting the encoding sequence for the AviTag™ (GLNDIFEAQKIEWHE, Avidity, Aurora, CO, USA) between those encoding for the TEV protease cleavage site and the N-CTD. This was achieved by amplifying the N-CTD plasmid using a pair of primers each containing part of the AviTag sequence and partially aligning with the template. (All the primers used for these cloning are listed in Suppl. Table S15). The nucleotide sequence of the three plasmids (N-FL, N-CTD and AT-N-CTD) was confirmed by Sanger sequencing.

Recombinant N-FL, N-CTD, and AT-N-CTD were expressed in *E. coli* BL21(DE3) pLysS cells by IPTG induction at 18 °C O/N. The cell pellet was then resuspended in lysis buffer (50 mM Tris–HCl, pH 8.0, 1 M NaCl, 1 mM DTT, 0.2 mg/ml PMSF, 10 µg/ml DNase I, 20 µg/ml RNase A, 5 mM MgCl2, cOmplete EDTA-free protease inhibitor (Roche), and 0.5 mg/ml lysozyme), and cells were disrupted by incubation at RT for 1 h followed by ultrasonication. The recombinant protein was purified from the clarified solution by Immobilized Metal Affinity Chromatography (IMAC) using a 5-ml HisTrap FF crude column (Cytiva) pre-equilibrated in 50 mM Tris–HCl, pH 8.0, 300 mM NaCl, 1 mM DTT, 20 mM Imidazole. The His-tagged protein was then eluted with 500 mM of imidazole. The His6-GST-tag was cleaved O/N by TEV protease under dialysis in 50 mM Tris–HCl, pH 8.0, 300 mM NaCl, 1 mM DTT, followed by reverse IMAC which allowed the recovery of the untagged protein into the column flow-through. This was further purified by a 5 ml HiTrap Heparin HP column (Cytiva) pre-equilibrated in 50 mM Tris–HCl, pH 8.0, 300 mM NaCl and 1 mM DDT. Bound N-protein was eluted with a NaCl linear gradient (0.3–1.0 M). A final polishing step was performed by Size Exclusion Chromatography, using HiLoad 16/600 Superdex 200 pg for N-FL, and HiLoad 16/600 Superdex 75 pg (Cytiva) for both N-CTD and AT-N-CTD. N-FL was eluted in 20 mM sodium phosphate, pH 7.2, 150 mM NaCl, while both N-CTD and AT-N-CTD were eluted in 25 mM sodium phosphate 25 mM, pH 6.0, 50 mM NaCl, 0.5 mM EDTA. The purity and integrity of the recombinant proteins was assessed by SDS‐PAGE analysis. Folding and stability was tested by 1D 1H-NMR and CD experiments.

### ELISA assay

ELISA assay was carried out on a 96-well multiwell plate, coated with either the N-FL protein or the N-CTD protein at a concentration of 5 µg/mL in the coating buffer (0.05 M Carbonate-Bicarbonate, pH 9.6). The negative control was performed plating the coating buffer solution. The plate was incubated O/N at 4 °C in a humid chamber. After removal of coating solution and plate washing with PBS buffer (pH 7.4), a blocking solution (4% non-fat dry milk-0.05% PBST) was added for 2 h at RT. The blocking solution was removed,100 µL/well of each scFv diluted in blocking solution at the concentration of 3 µg/mL were plated in duplicates and the plate was incubated O/N at 4 °C in a humid chamber. An unrelated scFv (anti Amyloid Beta) was used as negative control. After removal of the scFv and washing with PBS Tween 0.05%, the plate was incubated for 2–3 h at RT with 100 µL/well of MAb anti-V5 (1:1000, Invitrogen) in blocking solution, and then with 100 µL/well of secondary anti-mouse IgG-HRP conjugated (Peroxidase AffiniPure Goat Anti-Mouse IgG (H + L) Jackson ImmunoResearch, #115-035-003) 1:5000 in blocking solution for 1 h at RT. 100 µL/well of TMB solution (Sigma) was added to the plate and the colorimetric reaction was blocked by 100 µL/well of H2SO4 1 M solution. Two wells per each tested scFv were not challenged with MAb anti-V5, in order to exclude any cross-reaction with the secondary antibody. The colorimetric signal was read at a wavelength of 450 nm by a microplate reader (iMark™ Microplate Reader, Bio-Rad). All the results were blank-subtracted. All the scFv were tested after IEX-purification, before SEC step.

### Biotinylation of N-protein constructs

Biotin-labelling of N-FL and N-CTD was performed in vitro using the Thermo Scientific™ EZ-Link™ NHS-PEG4-Biotin reagent following the manufacturer’s protocol. Briefly, the crosslinking reaction between the N-hydroxysuccinimide ester (NHS) group of EZ-Link™ and lysine and N-terminal amino groups was conducted with a molar ratio 1:1 protein:EZ-Link™ for 2 h on ice. Excess of the linker was removed by 2 ml Zeba™ Spin Desalting Columns (7 K MWCO). For the AT-N-CTD construct, the presence of the AviTag™ allowed the biotinylation of the only N-terminal amine by enzymatic reaction with recombinant BirA ligase (Avidity, Aurora, CO, USA). The enzyme was added to 30 μM of Avi-tagged substrate in the presence of the supplied reaction mixture (SuperMix) containing biotin and ATP. The ligation reaction was carried out at RT for 1 h 30 min. Un-reacted biotin was then removed by loading the mixture onto a Superdex 75 10/300 GL column (Cytiva). Biotinylation was confirmed by successful loading of biotinylated AT-N-CTD onto BLI streptavidin biosensors. Concentration of biotinylated N protein was measured by UV–Vis spectrophotometry using absorbance at 280 nm and calculated molar absorption coefficient.

### Bio-layer interferometry assay

The Bio-layer interferometry (BLI) assay was employed to explore the binding affinity of recombinant anti-N scFvs that eluted as monomer in the SEC step. The assay was performed in black 96-well plates at 20 °C on ForteBio’s Octet® RED96e instrument using Streptavidin (SA) biosensors. Prior to use, biosensors were soaked for 10 min into the supplied kinetic buffer (PBS 1X, 0.02% Tween20, 0.1% BSA, 0.05% sodium azide, pH 7.4 – Forte-Bio). Biotinylated N protein was then loaded onto the biosensors by soaking these for 800 s into the protein solution at a concentration of 5 μg/mL for N-FL, 7.5 μg/mL for N-CTD, and 2.5 μg/mL for AT-NCTD. It followed the sensors quenching with 10 μg/ml Biocytin for 60 s, and equilibration in the kinetic buffer for 300 s prior to baseline collection. Recombinant scFv #24 and #336 were used as analytes in a twofold dilution series ranging from 31.25 to 250 nM. The experiment was designed in-column, with each sensor dipping a single analyte concentration. Association step was carried out for 600 s, to which followed the dissociation step for 300 s by incubation of the sensors onto kinetic buffer. The sequence was repeated with unloaded sensors as references. Binding constant (K_D_), association (k_a_) and dissociation (k_d_) rate constants values were estimated by globally fitting the BLI response intensity (nm) as a function of scFv concentration (nM) with the Octet Data Analysis Software, using a 1:1 Langmuir binding model (sensor unlinked).

### Cell lines

Vero-TMPRSS2 cells (kindly provided from San Raffaele Hospital, Milan) were cultured in DMEM (Gibco, Thermo Fisher, #11,695) or DMEM-F12 (Sigma, #D0697) culture medium supplemented with 10% FBS (Euroclone, #ECS0180LH), 1 mM L-glutamine (Sigma), 1 mM sodium pyruvate (Sigma), at 37 °C with 5% CO2.

### Isolation and titration of the SARS-CoV-2 B.1 strain

SARS-CoV-2 manipulation was performed in the biohazard safety level 3 (BSL3) facility of the Virology Unit, Pisa University hospital, Pisa, Italy, and in compliance with the European Committee and the World Health Organization laboratory biosafety guidelines. The SARS-CoV-2 strain used B.1 (hCoV-19/Italy/LOM-UniSR10/2021, GISAID Accession ID: EPI_ISL_2544194), was isolated from nasopharyngeal swab specimens of an infected individual. Briefly, Vero-TMPRSS2 cells were plated at approximately 80% confluence and infected with 500 µl of the nasal swab diluted in 5 ml of medium. Culture plates were incubated at 37 °C, 5% CO2, and shaken every 15 min for two hours. At the end, medium supplemented with 5% serum was added, and cells were cultured until a full cytopathic effect was achieved. Cells were then resuspended in a lysis buffer and centrifuged at 900 g for 10 min. The supernatants containing virus particles were filtered with a 0.45 µM strainer and aliquots were stored at -80 °C until use. Viral stocks were titered by limited dilution according to the Reed and Muench method^27^ and expressed as Tissue Culture Infectious Dose 50%/ml (TCID50/ml). The titer achieved was: B.1 7 × 10^8^ TCID50/ml.

### Transfection of VERO-TMPRSS2 cells with scFvs anti-N protein

10^6^ Vero TMPRSS-2 cells were seeded into d10 cell culture dishes; on the following day, cells were transfected with the scFvs cloned in the pEF1α-IRES-ZsGreen1 plasmid by using Lipofectamine LTX (thermo fisher, A12621). Briefly, transfection solution was prepared by mixing 600 µl of OPTIMEM + 20 µl of Lipofectamine with 600 µl of OPTIMEM containing 10 µl of plus reagent and 10 µg of DNA for each ScFvs. The solution was incubated 20 min before addition to the cell culture medium. Cells were incubated 48 h after transfection and then analysed by FACS to determine the percentage of transfection.

### Co-immunoprecipitation of anti-N scFvs and N Protein from Vero TMPRSS-2 cells co-expressing cells

1 × 10^6^ Vero TMPRSS-2 cells were plated in a 100 mm plate, transfected with scFvs anti-N protein by Lipofectamine LTX (Thermo Fisher, #A12621), as described above, and subsequently infected with SARS-CoV-2 B.1 strain at MOI 1. 48 h post-infection, cells were harvested and the resulting protein extracts were immunoprecipitated both with anti-N SARS-CoV-2 antibody and anti HA antibody. In detail, proteins were extracted in an ice-cold RIPA buffer supplemented with protease and phosphatase inhibitors (Sigma-Aldrich, #PPC1010). 500 μg of proteins were quantified using Bradford assay (Thermo Fisher Scientific, #23238). 100 μl of Pierce Protein A/G magnetic beads (Thermo Scientific, #88803) were incubated with 5 μg of primary HA antibody (Invitrogen, #MA5-27,915) for 20 min at RT. Proteins were incubated with the beads previously functionalized, and antibodies O/N at 4 °C. Samples were washed in TBS-Tween 0.05% and processed for WB experiments. Proteins were separated by SDS-PAGE (Biorad) and electro-blotted onto nitrocellulose membranes (Amersham). Membranes were blocked in a solution containing 5% milk in PBS-Tween 0.1% (v/v) for 1 h at RT. Primary antibodies were diluted in the blocking solution and were incubated O/N at 4 °C. Membranes were washed three times in PBS-Tween (0.1%) and then incubated at RT for 1 h with HRP-labeled secondary antibodies, diluted in the blocking solution. The used antibodies at the corresponding concentration are: HA Tag (Invitrogen, #MA5-27915) 1:1000; Actin (Invitrogen, #MA5-3313) 1:2000; SARS-CoV-2 Nucleocapsid (Genetex, #GTX135357) 1:1000; HRP-conjugated anti-mouse (Sigma-Aldrich, #A904); HRP-conjugated anti-rabbit (Sigma-Aldrich, #A0545).

### Supplementary Information

Supplementary Information.

## Data Availability

The datasets relative to the NGS sequencing of the 12 scFvs libraries generated during the current study are available in the Zenodo repository, with the following 10.5281/zenodo.10557297.
