# Author Correction: Selection and characterization of human scFvs targeting the SARS-CoV-2 nucleocapsid protein isolated from antibody libraries of COVID-19 patients

**DOI:** 10.1038/s41598-024-68554-w

**Published:** 2024-08-01

**Authors:** Simonetta Lisi, Francesca Malerba, Paola Quaranta, Rita Florio, Ottavia Vitaloni, Elisa Monaca, Bruno Bruni Ercole, Angela Rachel Bitonti, Olga del Perugia, Marianna Mignanelli, Paola Perrera, Rafaele Sabbatella, Francesco Raimondi, Carmen Rita Piazza, Anna Moles, Caterina Alfano, Mauro Pistello, Antonino Cattaneo

**Affiliations:** 1 Bio@SNS Laboratory, Scuola Normale Superiore, 56126 Pisa, Italy; 2 Fondazione EBRI (European Brain Research Institute) Rita Levi-Montalcini, 00161 Rome, Italy; 3 Structural Biology and Biophysics Unit, Fondazione Ri.MED, 90133 Palermo, Italy; 4 Retrovirus Centre, Department of Translational Research, University of Pisa, 56126 Pisa, Italy; 5 Virology Operative Unit, Pisa University Hospital, 56124 Pisa, Italy; 6 Department of Medical Biotechnologies, University of Siena, 53100 Siena, Italy; 7 Genomnia Srl, 20091 Bresso, MI Italy; 8 Institute of Biochemistry and Cell Biology, CNR, 80131 Napoli, Italy

Correction to: *Scientific Reports* 10.1038/s41598-024-66558-0, published online 09 July 2024

The Funding section in the original version of this Article was incomplete.

“This work was supported by the Regione Lombardia under Grant number 2014IT16RFOP012-POR FESR REGIONE LOMBARDIA 2014-2020-ASSE 1: AZIONEI.1. B.1.3 and by bando Ricerca Salute 2018 “Tuscany Antiviral Research Network (TUSCAVIR.NET)”.”

now reads:

“This work was supported by the Regione Lombardia under Grant number 2014IT16RFOP012-POR FESR REGIONE LOMBARDIA 2014-2020-ASSE 1: AZIONEI.1. B.1.3 and by bando Ricerca Salute 2018 “Tuscany Antiviral Research Network (TUSCAVIR.NET)”. This work was supported by the Open Access Publishing Fund of the Scuola Normale Superiore.”

The original Article has been corrected.

