# Supplementary material for: Selection and characterization of human scFvs targeting the SARS-CoV-2 nucleocapsid protein isolated from antibody libraries of COVID-19 patients
Source: Sci Rep. 2024 Jul 9;14:15864. doi: 10.1038/s41598-024-66558-0 (PMC11233501; doi:10.1038/s41598-024-66558-0)
Supplement: Supplementary file 1 — Supplementary Information. [file 41598_2024_66558_MOESM1_ESM.pdf]

SUPPLEMENTARY MATERIAL

Selection and characterization of a panel of human scFvs targeting the SARS-CoV-2 nucleocapsid protein isolated from antibody libraries of COVID-19 patients

Simonetta Lisi[a§], Francesca Malerba[b§], Paola Quaranta[d,e,] Rita Florio[b], Ottavia Vitaloni[a], Elisa Monaca[c], Bruno Bruni Ercole[b], Angela Rachel Bitonti[a], Olga del Perugia[a], Marianna Mignanelli[a], Paola Perrera[d], Raffaele Sabbatella[c] Francesco Raimondi[a], Carmen Rita Piazza[d,g], Anna Moles [h,i], Caterina Alfano[c], Mauro Pistello[d,e] and Antonino Cattaneo[a,b*].

[a]Bio@SNS Laboratory, Scuola Normale Superiore, 56126 Pisa, Italy

[b]Fondazione EBRI (European Brain Research Institute) Rita Levi-Montalcini, 00161, Rome, Italy

[c]Structural Biology and Biophysics Unit, Fondazione Ri.MED, Palermo 90133, Italy

[d]Retrovirus Centre, Department of Translational Research, University of Pisa, 56126 Pisa, Italy

[e]Virology Operative Unit, Pisa University Hospital, 56124 Pisa, Italy

[f]Institute of Neuroscience - CNR, 56124 Pisa, Italy

[g]Department of Medical Biotechnologies, University of Siena, 53100 Siena, Italy

[h]Genomnia srl, 20091 Bresso(MI), Italy,

[i]Institute of Biochemistry and Cell Biology, CNR, 80131 Napoli, Italy

[§]Co-first authors

*Corresponding author: antonino.cattaneo@sns.it

Library #	Sample	Age	Gender	Neutralising antibodies titer	Anti-S antibodies titer		RNA conc ug/ul	Absorbance ratio A260/280	RNA quality
					IgA	IgG EI			
	1	75	F	1/160	5.5	8.2	1,11	1,84	good
	2	62	M	1/160	1.1	12.2	0,343	1,84	poor
	3	47	M	1/40	2.8	6.6	0,57	1,68	poor
	4	60	M	1/40	n.a.	n.a.	0,446	1,91	medium
	5	32	F	1/80	n.a.	68 (SORIN)	0,913	1,79	medium
	6	50	M	1/80	3.9	6.1	0,011	1,62	no RNA
	7	40	M	1/80	0.1	7.7	0,369	1,98	medium
	8	60	M	1/80	0.9	4.9	1,37	1,6	poor
	9	51	M	1/160	5.6	9.0	0,683	1,98	good
	10	43	M	1/40	1.1	1.8	2,302	1,6	low
	11	52	M	1/40	1.7	1.8	2,826	1,59	low
	12	41	M	1/40	1.0	1.4	0,444	1,5	No RNA
	13	55	M	1/160	3.3	3.4	1,88	1,56	No RNA
	14	29	F	1/160	2.0	1.7	1,022	1,46	low
	15	37	M	1/160	0.9	2.9	3,724	1,85	No RNA
	16	34	F	1/80	1.5	1.8	2,204	1,69	low
	17	57	F	1/160	4.3	5.6	0,67	1,56	low
	18	33	F	1/160	1.9	3.3	1,924	1,62	No RNA
	19	45	M	1/320	6.2	9.0	1,537	1,64	low
	20	47	M	1/640	1.8	7.3	2,125	1,65	low
	21	41	M	1/160	1.1	4.1	0,928	1,68	low
**2**	**22**	**46**	**F**	**1/320**	**5.9**	**8.1**	**0,823**	**2.00**	**good**
	23	50	M	1/80	3.1	4.6	0,803	1,6	low
**6**	**24**	**48**	**M**	**1/160**	**>1.1**	**6.8**	**1,158**	**1,99**	**good**
**1**	**25**	**63**	**M**	**1/160**	**6.7**	**4.5**	**1,087**	**2.37**	**good**
	26	43	M	1/80	1.6	6.0	0,467	1,97	good
	27	36	M	1/40	0.1	2.6	0,646	1,97	good
	28	30	M	1/40	1.7	1.7	0,764	1,96	good
	29	39	F	1/40	2.1	2.3	0,707	1,96	good
	30	52	M	1/40	1.3	3.3	0,701	1,98	good
	31	35	M	1/80	5.0	3.9	0,857	1,99	good
	32	51	M	1/160	4.2	7.3	0,729	1,91	good
	33	58	M	1/40	0.7	2.2	0,523	1,92	good
	34	47	M	1/160	6.0	5.6	0,517	1,92	good
	35	35	M	1/40	2.2	1.6	0,533	1,84	good
	36	51	M	1/80	3.1	3.5	0,725	1,94	good
**3**	**37**	**53**	**M**	**1/160**	**4.0**	**5.6**	**0,716**	**1,93**	**good**
	38	57	M	1/80	2.9	4.7	0,806	1,94	good
**4**	**39**	**45**	**M**	**1/320**	**6.6**	**7.3**	**0.553**	**2.01**	**good**
**5**	**40**	**37**	**M**	**1/160**	**0.9**	**3.4**	**1.078**	**2.02**	**good**

Suppl. Table S1: Information of the 40 patients who had recovered from COVID-19 enrolled for biological samples collection of and PBMC isolation. Blood samples were collected by the

Virology Unit of the Azienda Ospedaliero Universitaria Pisana (AOUP) and screened for the presence of COVID-19 specific neutralising antibodies measured (see below for description). Anti S antibodies titer as measured using SARS-CoV-2 IgA and IgG ELISA kits® detecting IgA or IgG against a recombinant form of the S1 subunit (Euroimmun, Diagnostica Medica, Italia). In bold are highlighted the 6 patients from which the scFvs libraries (6 IgM and 6 IgG/IgA) were prepared.

Neutralisation assay: Patients' sera were tested in quadruplicate using 2-fold dilutions from 1:40 to 1:640 and compared with a control serum (1711). The control serum 1711 was selected amongst 2,000 sera tested for anti-SARS-CoV-2 neutralising activity and represented a high titer serum. The assay was performed by using an aliquot of SARS-CoV-2 B.1 (hCoV-19/Italy/LOM-UniSR10/2021, GISAID Accession ID: EPI_ISL_2544194) viral strain from titrated stocks. Tests were performed in 96-well plates containing 100 $TCID_{50}$/50 µl viral preparation per well that were incubated with 50 µl of each dilution of serum samples or controls in cell culture medium. This suspension was incubated at 37°C for 1 hour and then supplemented with 10,000 cells/100µ/well of Vero-TMPRSS2 cell lines. Plates were incubated for three days and then examined for cytopathic effect. A control plate was used for viral strain to verify the input titer.

	IgM			IgG/IgA		
	VH	VL		VH	VL	
Library#	Clonotypes without stop codon	Clonotypes without stop codon	Combinatorial diversity	Clonotypes without stop codon	Clonotypes without stop codon	Combinatorial diversity
1	544.395	67.573	3.68×10^{10}	227.891	115.767	2.63×10^{10}
2	240.526	40.297	9.69×10^{9}	127.895	48.455	6.19×10^{9}
3	305.118	51.069	1.55×10^{10}	180.035	51.650	9.29×10^{9}
4	98.304	49.871	4.90×10^{9}	131.745	31.412	4.13×10^{9}
5	441.762	60.907	2.69×10^{10}	321.196	106.497	3.42×10^{10}
6	278.163	83.972	2.33×10^{10}	464.001	108.306	5.02×10^{10}

Suppl. Table S2: **NGS libraries sequencing data**. For each type of library (IgM or IgG/IgA) are presented the number of individual clonotypes (comprising only those without a stop codon) for the VH and VL and the combinatorial diversity obtained by multiply the VH clonotypes for the and VL clonotypes.

	IgM			IgG/IgA		
	VH	VL		VH	VL	
Library#	Clonotypes with stop codon	Clonotypes with stop codon	Combinatorial diversity	Clonotypes with stop codon	Clonotypes with stop codon	Combinatorial diversity
1	596.108	68.720	4.09×10^{10}	254.299	122.825	3.12×10^{10}
2	273.714	40.791	1.11×10^{10}	151.323	52.227	7.90×10^{9}
3	342.119	51.853	1.77×10^{10}	208.128	52.706	1.09×10^{10}
4	101.263	53.785	5.44×10^{9}	155.835	32.050	4.99×10^{9}
5	482.495	62.248	$3. 00 \times 10^{10}$	355.848	113.033	4.02×10^{10}
6	308.796	85.608	2.64×10^{10}	502.384	114.876	5.77×10^{10}

Suppl. Table S3: NGS libraries sequencing data. For each type of library (IgM or IgG/IgA) are presented the number of individual clonotypes (comprising those with a stop codon) for the VH and VL and the combinatorial diversity obtained by multiply the VH clonotypes for the and VL colonotypes.

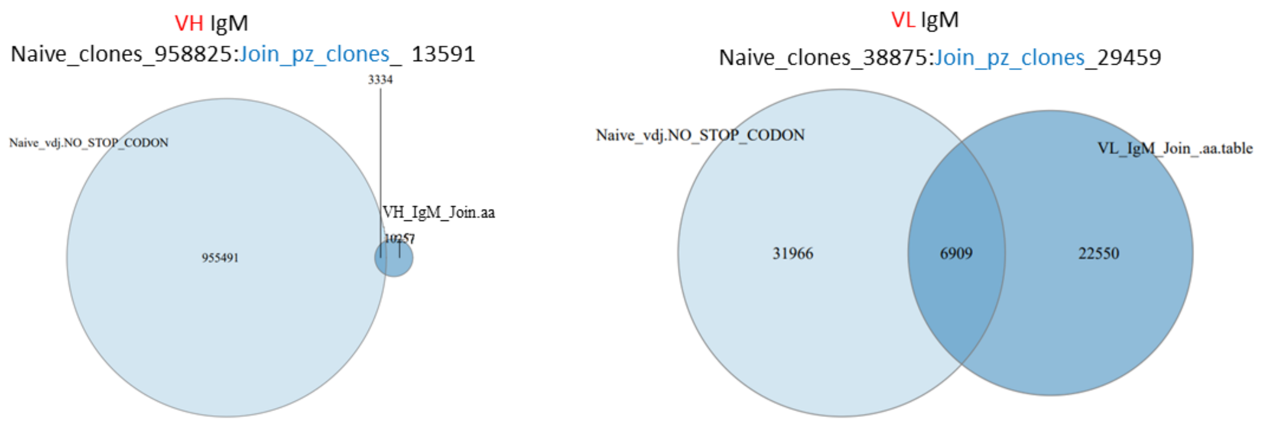

Suppl. Fig S1. Analysis of clonotypes overlap between a human naive IgM scFv library and the 6 IgM libraries from Covid-19 recovered patients.

Clonotypes were identified using the CDR3 amino acid sequence of the VH and VL

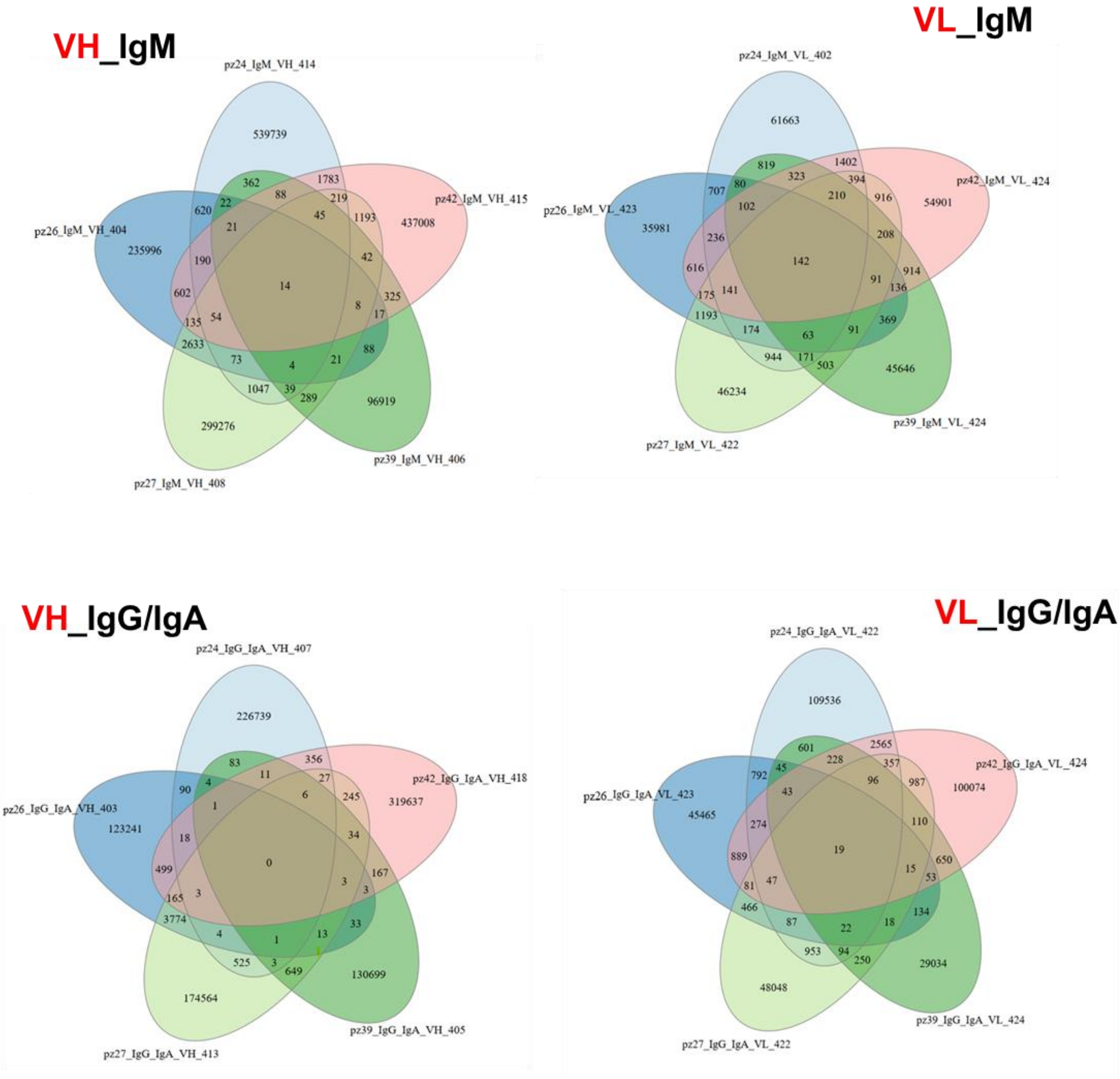

Suppl. Fig S2. Overlap occurrences of clonotypes between the VH and VL of the IgM and IgG/IgA libraries from Covid-19 recovered patients.

Top panels: Clonotypes occurrences in the VH (right panel) and in the VL clonotypes of the IgM libraries.

Bottom panels: Clonotypes occurrences in the VH (right panel) and in the VL clonotypes of the IgG/IgA libraries.

Clonotypes were identified using the nucleotide sequence of the CDR3

CLUSTAL 2.1 multiple sequence alignment

```
NCAP_SARS2        ---------MSDNGPQ-NQRNAPRITFGGPSDSTGSNQNGERSG----ARSKQRRPQGLP
NCAP_SARS         ---------MSDNGPQSNQRSAPRITFGGPTDSTDNNQNGGRNG----ARPKQRRPQGLP
NCAP_MERS1        ---------------MASPAAPRAVSFADNNDITNTNLSRGRG--------RNPKPRAAP
NCAP_CVHOC        MSFTPGKQSSSRASSGNRSGNG-ILKWADQSDQVRNVQTRGRRAQPKQTATSQQPSGGNV
NCAP_CVHN1        MSYTPGHYAGSRSSSGNRSGILKKTSWADQSERNYQTFNRGRKTQPKFTVSTQPQ--GNT
NCAP_CVHNL63      ---------------------MASVNWADDR---AARKK---------------------
NCAP_CVH22        ---------------------MATVKWADASEPQRGRQG---------------------
                                           .:..

NCAP_SARS2        NNTASWFTALTQHGK-EDLKFPRGQGVPINTNSSPDDQIGYYRRATRR-IRGGDGKMKDL
NCAP_SARS         NNTASWFTALTQHGK-EELRFPRGQGVPINTNSGPDDQIGYYRRATRR-VRGGDGKMKEL
NCAP_MERS1        NNTVSWYTGLTQHGK-VPLTFPPGQGVPLNANSTPAQNAGYWRRQDRK-INTGNG-IKQL
NCAP_CVHOC        VPYYSWFSGITQFQKGKEFEFVEGQGPPIAPGVPATEAKGYWYRHNRGSFKTADGNQRQL
NCAP_CVHN1        IPHYSWFSGITQFQKGRDFKFSDGQGVPIAFGVPPSEAKGYWYRHSRRSFKTADGQQKQL
NCAP_CVHNL63      FPPPSFYMPLLVSSDKAPYRVIPRNLVPIGKG-NKDEQIGYWN--VQERWRMRRGQRVDL
NCAP_CVH22        RIPYSLYSPLLVDSE-QPWKVIPRNLVPINKK-DKNKLIGYWN--VQKRFRTRKGKRVDL
                      * :  :    .     .   :  *:       .  **:    :   .   *   :*

NCAP_SARS2        SPRWYFYYLGTGPEAGLPYGANKDGIIWVATEGALNTPKDHIGTRNPANNAAIVLQLPQG
NCAP_SARS         SPRWYFYYLGTGPEASLPYGANKEGIVWVATEGALNTPKDHIGTRNPNNNAATVLQLPQG
NCAP_MERS1        APRWYFYYTGTGPEAALPFRAVKDGIVWVHEDGATDAP-STFGTRNPNNDSAIVTQFAPG
NCAP_CVHOC        LPRWYFYYLGTGPHAKDQYGTDIDGVYWVASNQADVNTPADIVDRDPSSDEAIPTRFPPG
NCAP_CVHN1        LPRWYFYYLGTGPYANASYGESLEGVFWVANHQADTSTPSDVSSRDPTTQEAIPTRFPPG
NCAP_CVHNL63      PPKVHFYYLGTGPHKDLKFRQRSDGVVWVAKEGAKTVNTSLGNRKRNQKPLEPKFSIALP
NCAP_CVH22        SPKLHFYYLGTGPHKDAKFRERVEGVVWVAVDGAKTEPTGYGVRRKNSEPEIPHFNQKLP
                   *: :*** ****     :    :*: **  . *          :

NCAP_SARS2        TTLPKGFYAEGSRGGSQASSRSSSRSRNSSRNSTPGSSRGTSP---ARMAGNGGDAALAL
NCAP_SARS         TTLPKGFYAEGSRGGSQASSRSSSRSRGNSRNSTPGSSRGNSP---ARMASGGGETALAL
NCAP_MERS1        TKLPKNFHIEGTGGNSQSSSRASSVSRNSSRSSSQGSRSGNSTRGTSPGPSGIGAVGGDL
NCAP_CVHOC        TVLPQGYYIEGS-GRSAPNSRSTSRTSSRASSAGSRSRANSGNRTPTSGVTPDMADQIAS
NCAP_CVHN1        TILPQGYYVEGS-GRSASNSRPGSRSQSRGPNNRSLSRSNSNFRHSDSIVKPDMADEIAN
NCAP_CVHNL63      PELSVVEFEDRSNNSSRASSRSSTRNNSRDSSRSTSRQQSRTRSDSNQSSSDLVAAVTLA
NCAP_CVH22        NGVTVVEEPD-----SRAPSRSQSRSQSRGRGESKPQSRNPSSDRNHNSQDDIMKAVAAA
                    :.     :     * . **. : . .   .       .

NCAP_SARS2        LLLDRLNQLESKMSGKGQQQQG---------------------QTVTKKSAAEASKKPRQ
NCAP_SARS         LLLDRLNQLESKVSGKGQQQQG---------------------QTVTKKSAAEASKKPRQ
NCAP_MERS1        LYLDLLNRLQALESGKVKQSQP---------------------KVITKKDAAAAKNKMRH
NCAP_CVHOC        LVLAKLGKDATKPQQVTKHT--------------------------AKEVRQKILNKPRQ
NCAP_CVHN1        LVLAKLGKDS-KPQQVTKQN--------------------------AKEIRHKILTKPRQ
NCAP_CVHNL63      LKNLGFDNQSKSPSSSGTSTPKKPNK----------------PLSQPRADKPSQLKKPRW
NCAP_CVH22        LKSLGFDKPQEKDKKSAKTGTPKPSRNQSPASSQTSAKSLARSQSSETKEQKHEMQKPRW
                  *    :..     .                                          * *

NCAP_SARS2        KRTATK--AYNVTQAFGRRGPEQTQGNFGDQELIRQGTDYKHWPQIAQFAPSASAFFGMS
NCAP_SARS         KRTATK--QYNVTQAFGRRGPEQTQGNFGDQDLIRQGTDYKHWPQIAQFAPSASAFFGMS
NCAP_MERS1        KRTSTK--SFNMVQAFGLRGPGDLQGNFGDLQLNKLGTEDPRWPQIAELAPTASAFMGMS
NCAP_CVHOC        KRSPNK--QCTVQQCFGKRGPN---QNFGGGEMLKLGTSDPQFPILAELAPTAGAFFFGS
NCAP_CVHN1        KRTPNK--HCNVQQCFGKRGPS---QNFGNAEMLKLGTNDPQFPILAELAPTPGAFFFGS
NCAP_CVHNL63      KRVPTR--EENVIQCFG---PRDFNHNMGDSDLVQNGVDAKGFPQLAELIPNQAALFFDS
NCAP_CVH22        KRQPNDDVTSNVTQCFG---PRDLDHNFGSAGVVANGVKAKGYPQFAELVPSTAAMLFDS
                  ** ..     .: *.**   *     *:*.  :   *..   :* :*:: *. .*::  *

NCAP_SARS2        RIGMEVTPSG-----------TWLTYTGAIKLDDKDPNFKDQVILLNKHIDAYKTFPPTE
NCAP_SARS         RIGMEVTPSG-----------TWLTYHGAIKLDDKDPQFKDNVILLNKHIDAYKTFPPTE
NCAP_MERS1        QFKLTHQNNDDHGNP-----VYFLRYSGAIKLDPKNPNYNKWLELLEQNIDAYKTFP---
NCAP_CVHOC        RLELAKVQNLSGNPDEPQKDVYELRYNGAIRFDSTLSGFETIMKVLNENLNAYQQDGMM
NCAP_CVHN1        KLDLVKRD---SEADSPVKDVFELHYSGSIRFDSTLPGFETIMKVLEENLNAYVNSNQNT
NCAP_CVHNL63      EVSTDEVGDN-----------VQITYTYKMLVAKDNKNLPKFIEQISAFTKPSSIKEMQS
NCAP_CVH22        HIVSKESGNT-----------VVLTFTTRVTVPKDHPHLGKFLEELNAFTR--EMQQHPL
                  ..                     : :   : .          :  :.
```

```
NCAP_SARS2       PKKDKKKKADETQALPQRQKKQQTVTLLPAADLDDFSKQLQQSMSSADSTQA--------
NCAP_SARS        PKKDKKKKTDEAQPLPQRQKKQPTVTLLPAADMDDFSRQLQNSMSGASADSTQA------
NCAP_MERS1       -KKEKKQKAPKEESTDQMSEPPKEHRVQGTQRTRTRPSVQPGPMIDVNTD----------
NCAP_CVHOC       N---MSPKPQRQRGHKNGQGENDNISVAVPKSRVQQNKSRELTAEDISLLKKMDEPYTED
NCAP_CVHN1       DSDSLSSKPQRKRGVKQLPEQFDSLNLSAG----TQHISNDFTPEDHSLLATLDDPYVED
NCAP_CVHNL63     QSSHVAQNTVLNASIPESKPLADDDSAIIEIVNEVLH-----------------------
NCAP_CVH22       LNPSALEFNPSQTSPATAEPVRDEVSIETDIIDEVN------------------------


NCAP_SARS2       ----
NCAP_SARS        ----
NCAP_MERS1       ----
NCAP_CVHOC       TSEI
NCAP_CVHN1       SVA-
NCAP_CVHNL63     ----
NCAP_CVH22       ----
```

Suppl. Fig S3. Multiple comparison of the mutations Nucleocapsid protein of representative Coronaviruses.

Nucleocapsid protein sequences of the human coronaviruses Sars-CoV-2, Sars-CoV, Mers, OC43, HKU1, NL63, 229E were aligned by Crustalw. The dimerization domain of Sars-cov2 is highlighted in cyan-

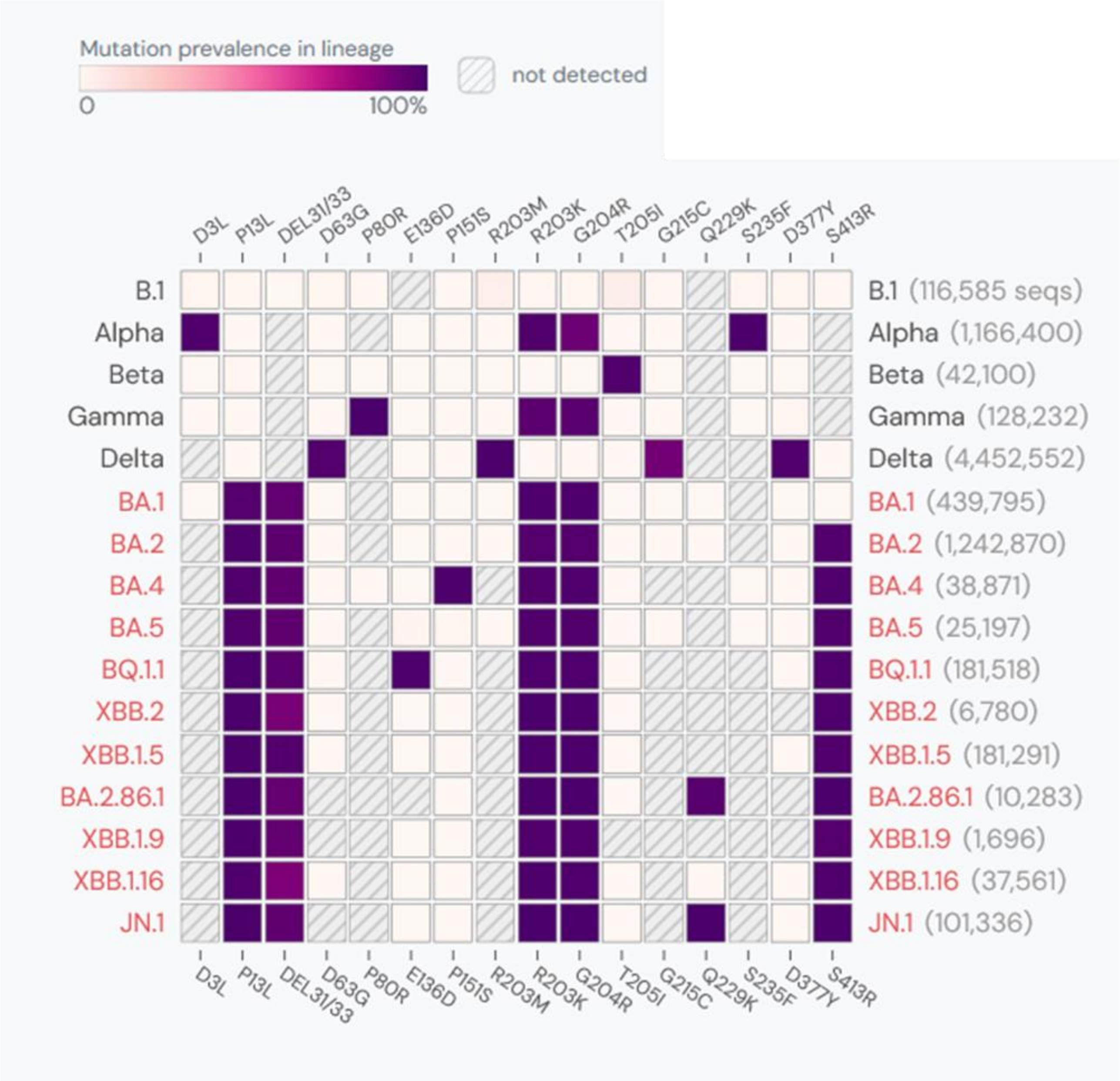

Suppl. Fig S4. Mutation prevalence in Nucleocapsid protein across lineages of 16 SARS-CoV-2 representative variants of concern.

By the means of the tool of lineage comparison provided by the website outbreak.info[1,2], we have compared the mutation prevalence in the Nucleocapsid sequences of the following variants of concern: B.1 Wuhan, B.1.17 (Alpha), B.1.351 (Beta), P1 (Gamma), B.1.617.2 (Delta), BA.1, BA.2, BA.4, BA.5, BQ.1.1 (Cerberus), XBB.2 (Gryphon), XBB.1.5 (Kraken), BA.2.86.1 (Pirola), XBB.1.9, XBB.1.16 (Arcturus), JN.1 and found that that no mutations fall in the CTD region (258-363 aa). In the upper part of the figure, the colorimetric legend of the mutation prevalence is indicated, while in the brackets the number of sequences analysed is shown.

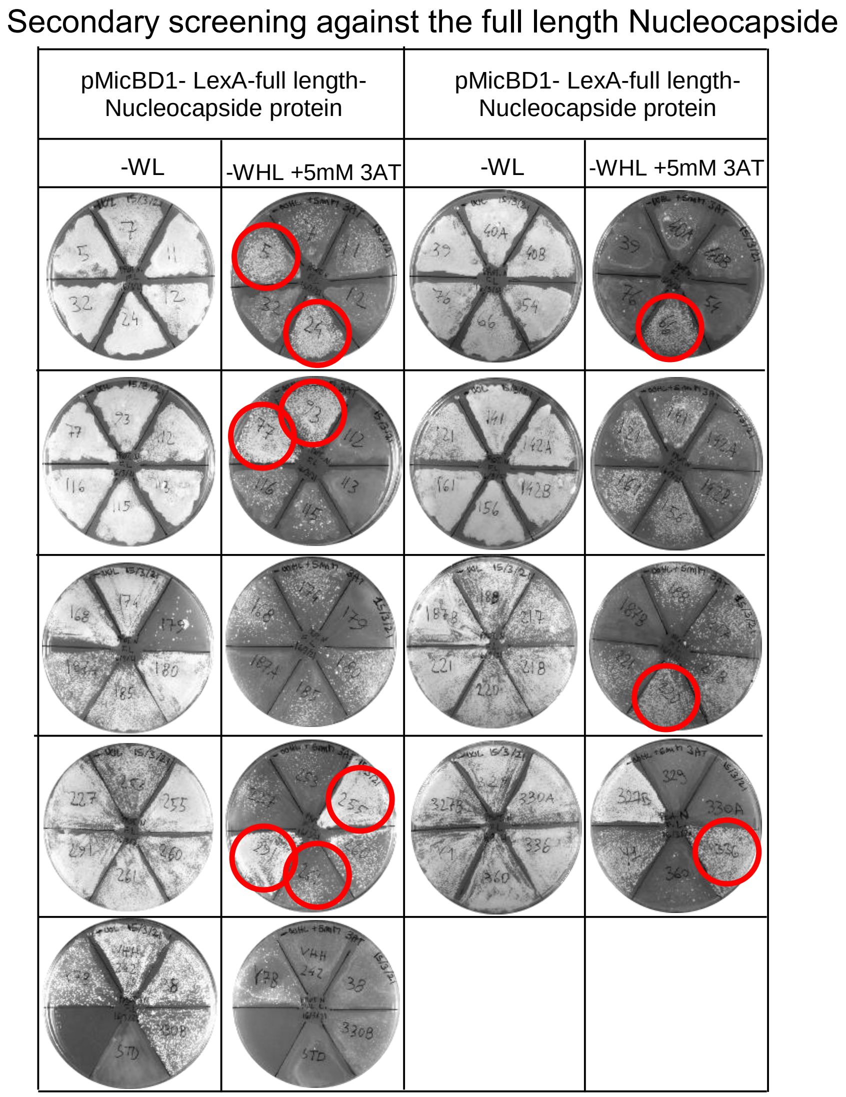

Suppl. Fig S5: Secondary screening of the selected scFvs against the full length Nucleocapsid protein.

The yeast strain expressing the LexA- full length Nucleocapsid of SARS-CoV2 (aa 1-419) was tested with the individual scFv retrieved from the primary screening. Clones 5, 24,66,77,93,220,255,261,291 and 336 (circled in red) showed a strong interaction with the bait protein LexA- full length Nucleocapsid, showing a high growth rate on the selective –WHL+5mM3AT plates. –WL: plates without Tryptophan and Leucine (to detected efficiency of transformation); –WHL+5mM3AT: plates without Tryptophan, Leucine and Histidine + 5mM 3 Amino-Triazole (to detected bait-prey interaction)

scFv #	VH CDR3 sequence	CDR3 lenght	VL CDR3 sequence	CDR3 lenght
5	CARGGWRYPNLGYYSYSYMDV	21	CQQYYSYPLT	10
24	CARGGYTVTKPIPVNWYFDL	20	CQQSYSTPYT	10
66	CARGGWARAARPIYYMDV	18	CQQADSFSIT	10
77	CARGGGTLLRGVMEAKWFDP	20	CASYTSSNIVL	11
93	CARDWWCTGDCFKYLYYGLDV	21	CAAWDDSLSGVV	12
220	GGWVRGARGHGYHSMDV	17	QAWDSSTVV	9
255	CTRGGYSNSGARFYGMDV	18	CQSADSSGTYV	11
261	CARGGWRYPNLGYYSYSYMDV	21	CAAYTSSLTVL	11
291	CARAGWKYADLYYQYSGMDV	20	CCSYAGSSTVV	11
336	CARGGWGTSHGGFYFHY	17	CQQSYSIPFTF	11

Suppl. Table S4. Amino acid sequence of the complementarity determining region 3 (CDR3) of the VH and VL of each of the selected anti-N scFvs.

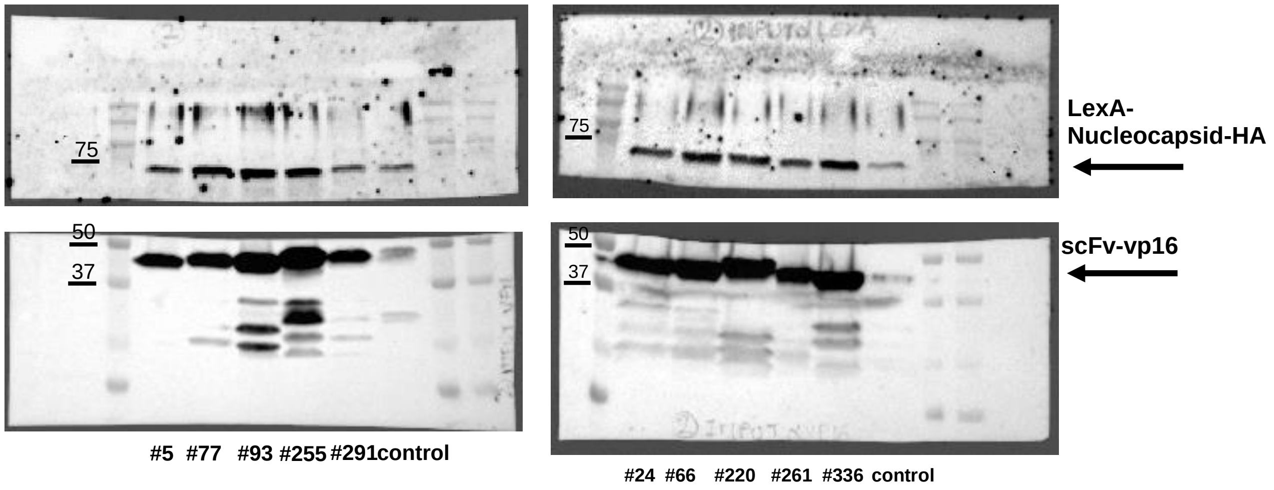

Supp. Fig S6. Input of the co-immunoprecipitation of Nucleocapsid full length protein with scFv anti Nucleocapsid in yeast shown in Fig 1B.

Protein extracts (100ug) from yeast strains expressing both the LexA-N-FL-HA protein (69 KDa) and each of the anti N scFvs-VP16 (5,77,93,255,291,24,66,220,261,336) or an unrelated scFv (control) have been immunoblotted with anti LexA antibody (top panels). The scFvs (~38.5 KDa) were detected with the anti VP16 antibody (bottom panels).

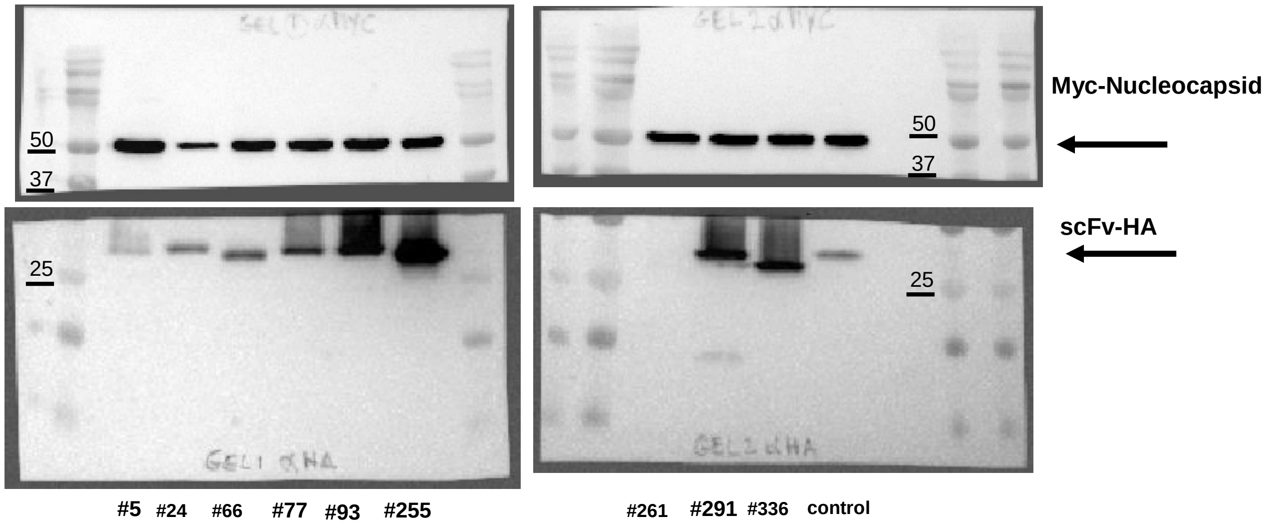

Supp. Fig S7. Input of the co-immunoprecipitation of Nucleocapsid full length protein with scFv anti Nucleocapsid in yeast shown in Fig 1C.

Protein extracts (80ug) of HEK 293T expressing both the Myc-N-FL protein (46.8 KDa) and the individual scFvs-HA (5, 24,66,77,93,255,291,336 or an unrelated scFv used as control) have been immoblotted with an anti Myc antibody to detect Nucleocapsid protein (top panel) or with an anti HA antibody to detect the scFvs (~ 28 KDa) (bottom panel).

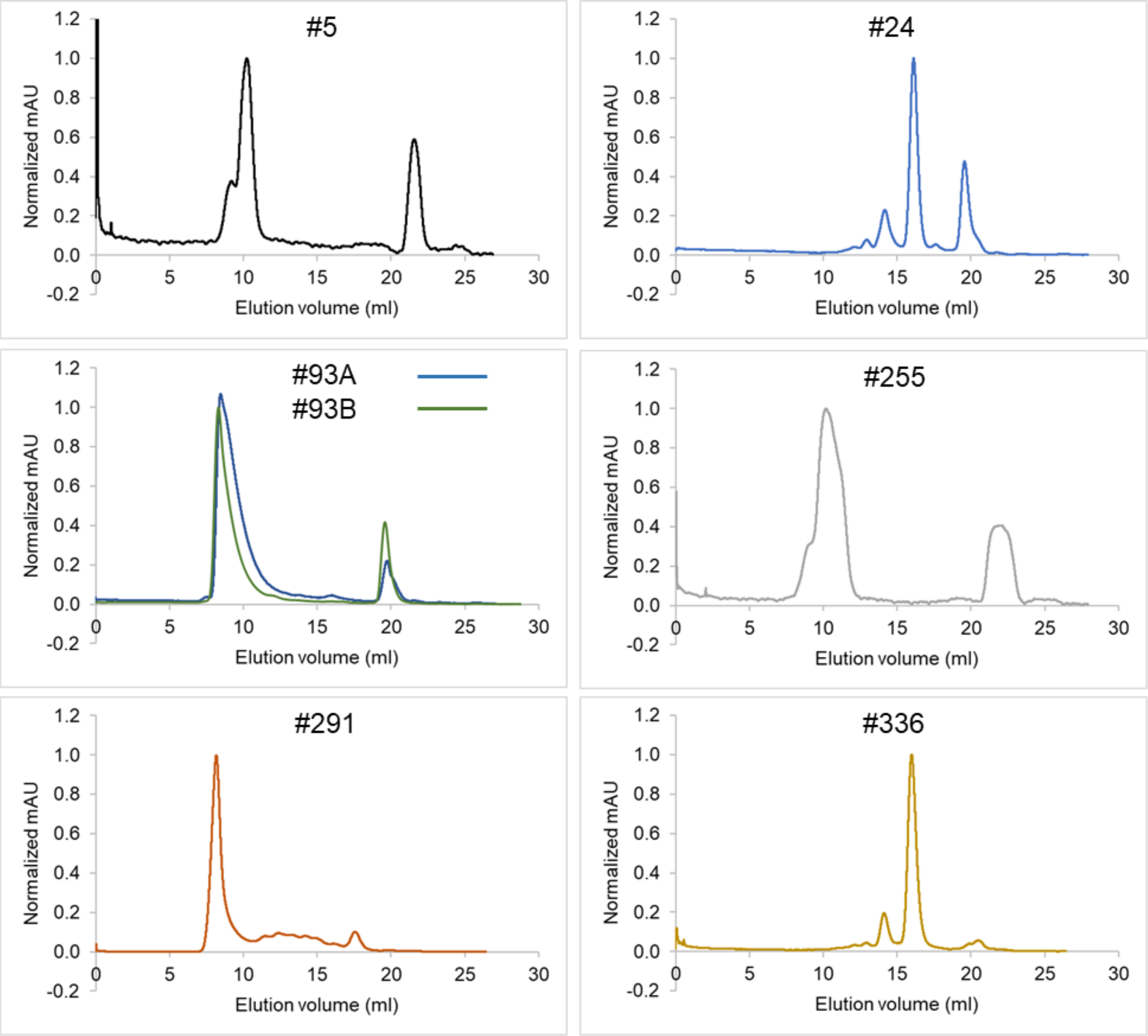

Suppl. Fig. 8. **Size exclusion chromatography profiles of recombinant scFvs**. The quaternary structure of recombinant scFvs, and in particular their property of being predominantly in the monomeric form, was tested by SEC analysis. ScFvs #24 and #336 exhibited a good yield and purity, with the monomeric form eluting around 15ml in accordance to the expected MW of 29.5 KDa. On the other hand, ScFvs #5, #93, #255, and #291 showed a high tendency to oligomerize, with almost all protein eluting at the column void volume. ScFvs #93 preparation was divided into fractions A, B: see Supplementary Fig. S23 and S24 for details.

Dot Blot Assay

Dot Blot Assay was carried out by spotting different concentrations of a recombinant SARS-CoV-2 N protein (RayBiotech) (1 µg, 0.5 µg, 0.25 µg, 0.125 µg, and 0.0625 µg.) in the fixed volume of 1 µL on a nitrocellulose membrane (GE Healthcare). The same volume of proNGF at the concentration of 1.4 µg/µL was spotted as negative control.

The membrane was set in TBS for 3 minutes and incubated for 2 hours and half in agitation with blocking solution (10% non-fat dry milk in 0.05% TBS-Tween (TBST)). The membrane was then incubated overnight at 4°C in 5% blocking solution containing 5 µg/mL of scFv.

After removal of the scFv and washing with 0.05% TBST, the membrane was incubated with a mouse MAb anti-V5 (Invitrogen) at the concentration 1:1000 for 2 hours at room temperature (RT) and then with the secondary antibody, an anti-mouse IgG HRP-conjugated (Peroxidase AffiniPure Goat Anti-Mouse IgG (H+L) Jackson ImmunoResearch) at the concentration 1:7000, for further 2 hours at RT. Spots on the membrane were detected by using ECL (GE healthcare) and an Invitrogen™ iBright™ Imaging System.

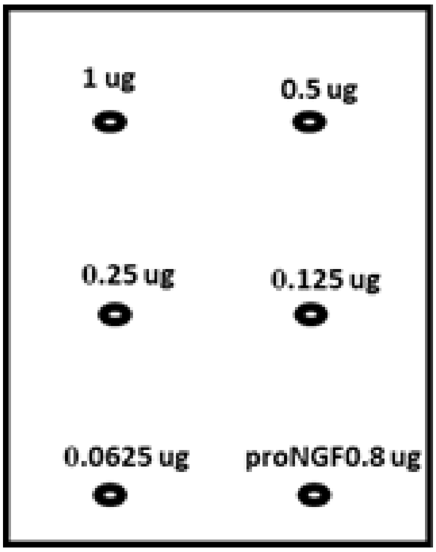

Suppl. Fig S9. Reference frame of the N protein loaded on the Dot Blot membrane. ProNGF represents the negative control.

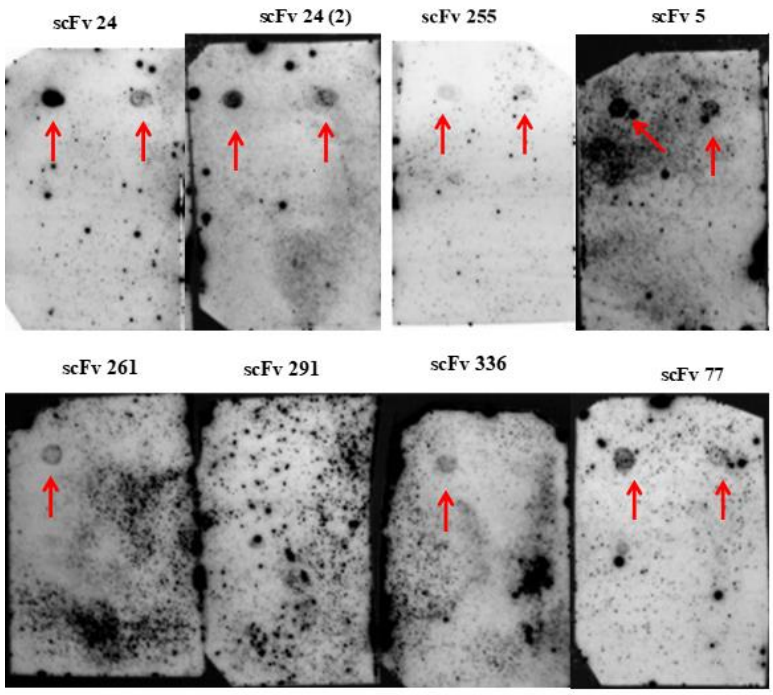

Suppl. Fig S10. Representative Dot Blots carried out on the scFv indicated on the top of the image. (The red arrows indicate binding-positive spots).

As shown in Suppl. Fig S10, scFvs 24, 255, 5 and 77 were able to recognize the two higher concentrations of the N protein, scFvs 261 and 336 bound N protein only at the highest concentration, while scFv 291 gave no signal, under the tested conditions.

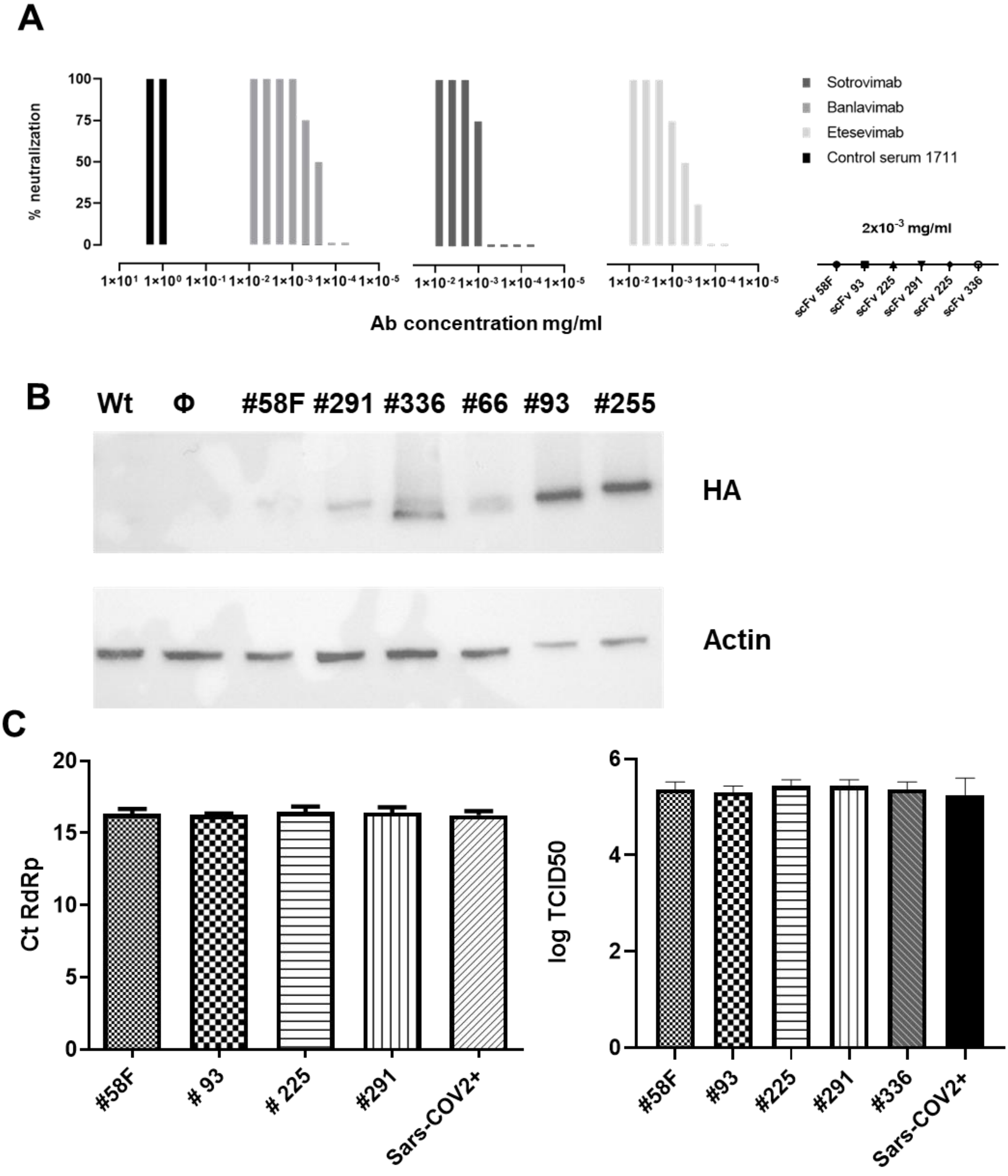

Suppl. Fig. S11. Lack of neutralising activity by anti-N scFvs as recombinant protein (A) and as intracellularly expressed (B, C). A) Comparison of activity of scFv anti-N protein, control serum and anti spike MAbs used in clinics[3]. All purified recombinant scFvs were used either alone or in combination, at concentrations ranging from 2000 ng/ml up to 0.2 ng/ml and compared with a positive-control serum obtained from a convalescent COVID-19 patient and with the anti-S MAbs Bamlanivimab, Etesevimab, and Sotrovimab[3]. **B**). Anti-N scFvs expression in Vero-TMPRSS2 cells.

scFvs cloned in the pEF1α-IRES-ZsGreen1 plasmid were expressed in Vero-TMPRSS2 cells. The scFvs-HA tagged (28 KDa) or an unrelated scFv (#58F), were detected using an anti HA antibody. Untransfected wild type cells (Wt) and cells transfected with the empty plasmid (Φ) cell extracts were loaded as negative control (upper panel); actin was used as loading control (lower panel). **C**) Vero-TMPRSS-2 cells were first transfected with the anti-N scFv (cloned in the pEF1α-IRES-ZsGreen1 plasmid) and 48 h post transfection, wild-type cells and cells expressing scFvs-HA were infected with the SARS-CoV-2 B.1 strain at MOI 0.1. The supernatants from the infected cells were harvested 48h post infection. The viral titer was expressed as threshold cycle (Ct) of viral RNA polymerase (RdRp) obtained by real time PCR, in the right graph the viral titer was expressed as logarithm of tissue culture infectious dose 50 (TCID50), i.e. the dilution of a virus required to infect 50% of a cell culture. No statistically significant reduction in virus titer was detected in any sample compared to the control.

Infection with SARS-CoV-2, B.1 strain, of cells transfected with scFvs anti-N protein

Vero TMPRSS-2 cells plated in d10 dishes and transfected with scFvs were subsequently infected with SARS-CoV-2 B.1 strain at multiplicity of infection (MOI) 0.1 to assess the ability of cells to neutralise the virus. Briefly, the culture medium was removed and cells were inoculated with the virus diluted in a fresh medium supplemented with 5% of FBS. Cells were incubated at 37°C for 3 h. After inoculum, cells were washed with PBS1x and then fresh culture medium with 5% of FBS was added. 48 h post infection, supernatants of infected cells were collected and titrated both by limited dilution as described above and by Real Time PCR.

Neutralisation assay

Selected recombinant anti-N scFvs (#5, 24, 77, 255, 261, 336, 291) and a non-related scFv (#58F) were tested individually or in various combination (#5,24; #77,255; #261,336; #291, 255; #291,336; #261,77; #5,77,24,255; #5,261,336,291) in quadruplicate in a neutralisation assay using 10-fold dilutions from 2000 ng/ml up to 0.2 ng/ml and were compared with the control serum 1711, obtained from a convalescent COVID patient, and with the anti-S monoclonal antibodies (MAbs) Bamlanivimab, Etesevimab, and Sotrovimab[3], obtained from clinical preparations and diluted in physiological solution to therapeutic concentrations. The control serum 1711 was selected amongst 2,000 sera tested for anti-SARS-CoV-2 activity and represented a high titer serum. The serum sample was tested in quadruplicate using 2-fold dilutions from 1:40 to 1:5,120; the MAbs were tested in quadruplicate using 2-fold dilutions from 8 µg/ml. The assay was performed by using an aliquot of B.1 viral strain from titrated stocks. Each aliquot was used only once. Tests were performed in 96-well plates containing 100 $TCID_{50}$/50 µl viral preparation per well that were incubated with 50 µl of scFvs samples or controls diluted in a cell culture medium. This suspension was incubated at 37°C for 1 hour and then supplemented with 10,000 cells/100µ/well of Vero-TMPRSS2 cell lines. Plates were incubated for three days and then examined for cytopathic effect. A control plate was used for viral strain to verify the input titer.

Western blot of VERO-TMPRSS2 cells transfected with scFvs anti-N protein

$3x10^5$ Vero-TMPRSS2 cells were plated in a 6-well plate and transfected the next day with the scFvs cloned in the pEF1α-IRES-ZsGreen1 plasmid using Lipofectamine LTX (Invitrogen, Thermo Fisher, Italy) according to manufacturer's protocol. Cells were collected 48h after transfection and lysed in an ice-cold RIPA buffer containing protease and phosphatase inhibitor cocktails (Sigma-Aldrich). Total protein extract was obtained by O/N incubation at 4 °C followed by centrifugation at 12000 rpm for 15 min at 4 °C. Proteins were quantified by Bradford assay (Thermo Fisher Scientific). 30 µg of protein lysate were mixed with 5X Laemmli Buffer and denatured at 70 °C for 10 min. Proteins were separated by SDS-PAGE (Biorad) and electro-blotted onto nitrocellulose membranes (Amersham). The membranes were blocked in a solution containing 5% milk in PBS-Tween 0.1% (v/v) for 1h at RT. Primary antibodies were diluted in the blocking solution and were incubated O/N at 4°C. Membranes were washed three times in PBS-Tween (0.1%) and then incubated at RT for 1h with HRP-labeled secondary antibodies, diluted in the blocking solution. The used antibodies at the corresponding concentration are: HA Tag (Invitrogen, #MA5-27915) 1:1000; Actin (Invitrogen, #MA5-3313) 1:2000; SARS-CoV-2 Nucleocapsid (Genetex, #GTX135357) 1:1000; HRP-conjugated anti-mouse (Sigma-Aldrich, #A904); HRP-conjugated anti-rabbit (Sigma-Aldrich, #A0545).

Real time PCR of SARS-CoV-2

SARS-CoV-2 RNA relative amounts detected as a cycle threshold (Ct) value for each experimental condition were compared, with a mean Ct value determined for the positive infection control. The viral RNA was purified from 100 µL of cell-free culture supernatant for each condition, using the QIAamp Viral RNA Mini Kit (Qiagen). The purified RNA was then used to synthesize first-strand complementary DNA, using the SuperScript First-Strand Synthesis System for RT-PCR (Thermo Fisher Scientific). To detect the complementary DNA, real-time PCR was performed using the SYBR Green dye–based PCR amplification and detection method. The SYBR Green PCR Master Mix (Thermo Fisher Scientific) was used, with the forward primer N SARS-CoV-2 fwd: TTACAAACATTGGCCGCAAA, and the reverse primer N SARS-CoV-2 rev: GCGCGACATTCCGAAGAA. The PCR conditions were: 95°C for 2 min, 45 cycles of 95°C for 20 s, annealing at 55°C for 20 s and elongation at 72°C for 30 s, followed by a final elongation at 72°C for 10 min. RT-PCR was performed using the ABI-PRISM 7900HT Fast Real Time instrument (Applied Biosystems) and optical-grade 96-well plates. Samples were run in duplicate, with a total volume of 20 µL.

Construction of human scFvs libraries

A scFv antibody library was assembled from the IgM cDNA and from the IgG/IgA cDNA (prepared from the PBMCs isolated from each of the 6 COVID19 recovered patients previously selected) following a protocol modified from Marks and Bradbury[4] and detailed in Fantini et al.[5].

IgM or IgG/IgA cDNA was used as a template to amplify VH and VL regions. Primers are designed to anneal to the external framework regions of the V genes. All the VH and VL subclasses were amplified together in a single reaction (one reaction for the VH, one for the light Vλ and one for the Vκ) using a mix of the 5' and 3' primers available (see below). Construction of each library occurred in five steps as shown in Suppl. Fig. S10 : i) amplification of VHs, Vκs and Vλs from cDNA; ii) construction of a linker (G4S)3 with primers specific for VHs, Vκs and Vλs; iii) assembly of each variable class (VHs, Vκs and Vλs) with the specific (G4S)3 linker (VH, Vκ and Vλ blocks); iv) pullthrough of VH block with Vκ blocks and VH blocks with Vλ blocks to generate scFv objects, and addition of restriction sites for BssHII at 5' and for NheI at the 3' to the scFv pullthrough products; v) Ligations of BssHII/NheI digested pullthroughs to vector pLinker220.

In the first step of the process, a mix of the 6 primers for the 5' and of the 4 primers for the 3' was used in a single reaction to amplify all VH subclasses concomitantly. Similarly, a mix of the 6 primers for the 5' and of the 5 primers for the 3' was used to amplify Vκs and a mix of the 7 primers for the 5' and of the 3 primers for the 3' was used to amplify Vλs., 6 primers for the 5' and 4 primers for the 3' were used to amplify VHs, 6 primers for the 5' and 5 primers for the 3' were used to amplify Vκs and 7 primers for the 5' and 3 primers for the 3' were used to amplify Vλs (see list below). For the second step, a 45 bp linker (G4S)3 was amplified from a pre-existing plasmid using a set of primers, with the 3' region annealing on the linker, and different protruding 5', overlapping perfectly either with VH framework4 or VL framework1. At this point, all classes individually amplified were mixed in equimolar ratio for VHs, Vκs and Vλs, and the mix used in the third step in a PCR to join V products to each V specific linker. The fourth step was performed joining VH blocks and VL blocks in a final pullthrough PCR (in which the linkers overlap) to produce the scFv. In this step the first 10 cycles were run without primers allowing the joining of the different blocks. Then, primers for the 5' of VHs bearing restriction site for BssHII and primers for the 3' of Vκs or Vλs bearing restriction site for NheI were added to the mix, generating the final ~750bp scFv product. Finally the pullthrough products were digested with BssHII/NheI enzymes and ligated to the BssHII/NheI digested vector pLinker220[6]

~1µg of ligation product was transformed by electroporation in Max Efficiency DH5α (Invitrogen). Transformation efficiency was assessed by plating on selective media serial dilution of the transformation and counting surviving colonies. The theoretical maximum of the library complexity was 1.58 x10^7. Transformed bacteria were inoculated in LB-SeaPrep Agarose (Lonza Rockland, Inc.) as described in Elsaesser[7]. Plasmids were extracted with Qiagen Plasmid Giga Kit.

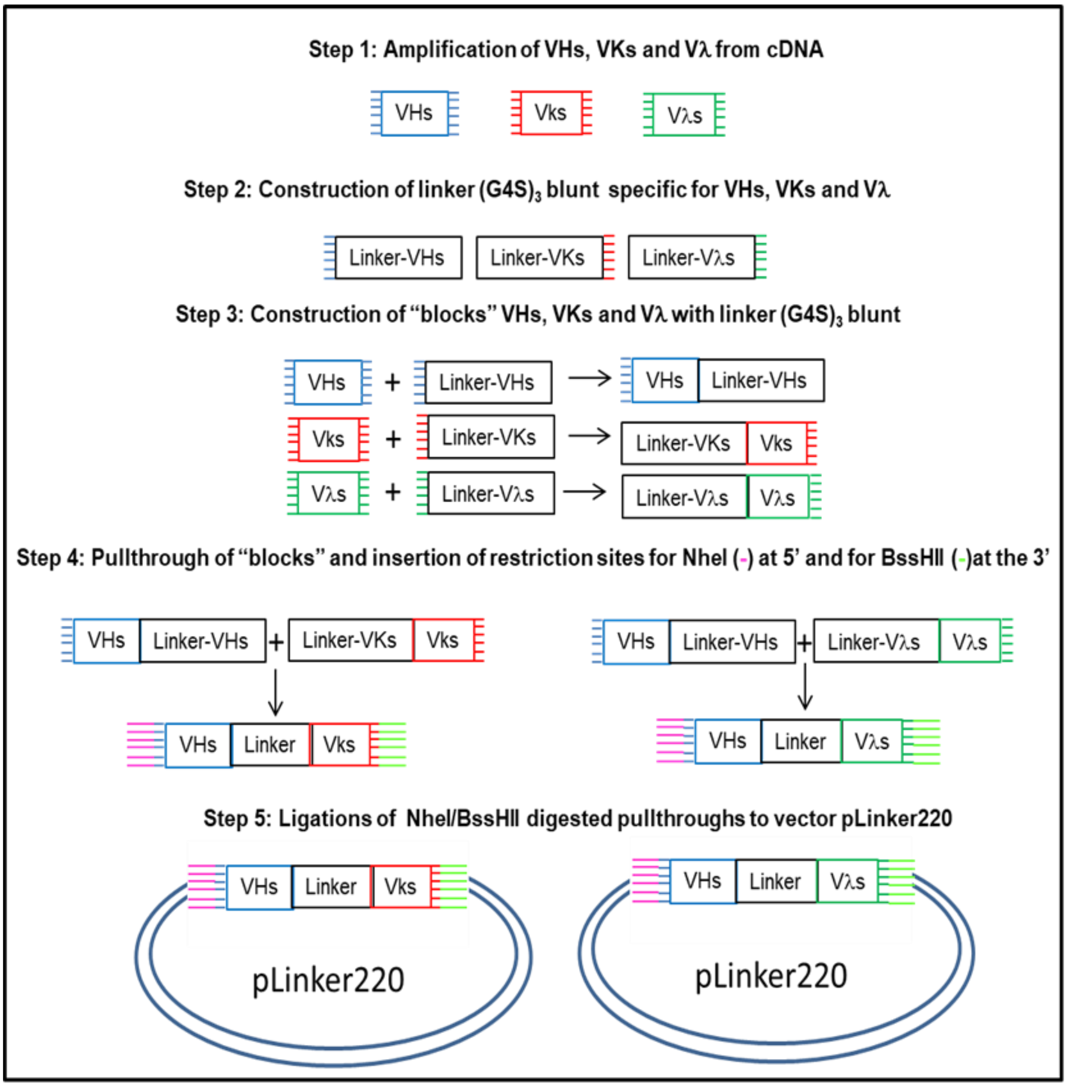

Suppl. Fig S12: Steps for recombinant scFv library assembly

Primers used for library construction

Primers for VH

BssHII-HuVH1aBACK: 5' gCCgcgcgcatgccCAGGTGCAGCTGGTGCAGTCTGG 3'

BssHII-HuVH2aBACK: 5' gCCgcgcgcatgccCAGGTCAACTTAAGGGAGTCTGG 3'

BssHII-HuVH3aBACK: 5' gCCgcgcgcatgccGAGGTGCAGCTGGTGGAGTCTGG 3'

BssHII-HuVH4aBACK: 5’gCCgcgcgcatgccCAGGTGCAGCTGCAGGAGTCGGG 3’

BssHII-HuVH5aBACK: 5’ gCCgcgcgcatgccGAGGTGCAGCTGTTGCAGTCTGC 3’

BssHII-HuVH6aBACK: 5’ gCCgcgcgcatgccCAGGTACAGCTGCAGCAGTCAGG 3’

HuJH1–2FOR: 5’ TGAGGAGACGGTGACCAGGGTGCC 3’

HuJH3FOR: 5’ TGAAGAGACGGTGACCATTGTCCC 3’

HuJH4–5FOR: 5’ TGAGGAGACGGTGACCAGGGTTCC 3’

HuJH6FOR: 5’ TGAGGAGACGGTGACCGTGGTCCC 3’

Primers for hVH “nanobody” library

NheI-HuJH 1–2 FOR : 5’ CGGCCGCGCTAGCTGAGGAGACGGTGACCAGGGTGCC 3’

NheI-HuJH 3 FOR: 5’CGGCCGCGCTAGCTGAAGAGACGGTGACCATTGTCCC 3’

NheI- HuJH 4–5 FOR: 5’CGGCCGCGCTAGCTGAGGAGACGGTGACCAGGGTTCC 3’

NheI-HuJH 6 FOR: 5’CGGCCGCGCTAGCTGAGGAGACGGTGACCGTGGTCCC 3’

Primers for Vk

HuVκ1aBACK: 5’ GACATCCAGATGACCCAGTCTCC 3’

HuVκ2aBACK: 5’ GATGTTGTGATGACTCAGTCTCC 3’

HuVκ3aBACK: 5’ GAAATTGTGTTGACGCAGTCTCC 3’

HuVκ4aBACK: 5’ GACATCGTGATGACCCAGTCTCC 3’

HuVκ5aBACK: 5’ GAAACGACACTCACGCAGTCTCC 3’

HuVκ6aBACK: 5’ GAAATTGTGCTGACTCAGTCTCC 3’

NheI-HuJκ1FOR: 5’ CGGCCGCgctagcACGTTTGATTTCCACCTTGGTCCC 3’

NheI-HuJκ2FOR: 5’ CGGCCGCgctagcACGTTTGATCTCCAGCTTGGTCCC 3’

NheI-HuJκ3FOR: 5’ CGGCCGCgctagcACGTTTGATATCCACTTTGGTCCC 3’

NheI-HuJκ4FOR: 5’ CGGCCGCgctagcACGTTTGATCTCCACCTTGGTCCC 3’

NheI-HuJκ5FOR: 5’ CGGCCGCgctagcACGTTTAATCTCCAGTCGTGTCCC 3’

Primers for Vλ

HuVλ1BACK: 5’ CAGTCTGTGTTGACGCAGCCGCC 3’

HuVλ2BACK: 5’ CAGTCTGCCCTGACTCAGCCTGC 3’

HuVλ3BACK: 5’ TCCTATGTGCTGACTCAGCCACC 3’

HuVλ3bBACK: 5’ TCTTCTGAGCTGACTCAGGACCC 3’

HuVλ4bBACK: 5’ CACGTTATACTGACTCAACCGCC 3’

HuVλ5BACK: 5’ CAGGCTGTGCTCACTCAGCCGTC 3’

HuVλ6BACK: 5’ AATTTTATGCTGACTCAGCCCCA 3’

NheI-HuJλ1FOR: 5’ CGGCCGCgctagcACCTAGGACGGTGACCTTGGTCCC 3’

NheI-HuJλ2-3FOR: 5’ CGGCCGCgctagcACCTAGGACGGTCAGCTTGGTCCC 3’

NheI-HuJλ4-5FOR: 5’ CGGCCGCgctagcACCTAAAACGGTGAGCTGGGTCCC 3’

Primers for Linkers

PlusLinker(G4S)3 : 5’ GGTGGAGGCGGTTCAGGCGGAG 3’

MinusLinker(G4S)3: 5’ CGATCCGCCACCGCCAGAGCCAC 3’

RHuJH1–2: 5’ GCACCCTGGTCACCGTCTCCTCAGGTGG 3’

RHuJH3: 5’ GGACAATGGTCACCGTCTCTTCAGGTGG 3’

RHuJH4-5: 5’ GAACCCTGGTCACCGTCTCCTCAGGTGG 3’

RHuJH6mod: 5’ GGACCACGGTCACCGTCTCCTCAGGTGG 3’

RHuVκ1aBACKFv: 5’ GGAGACTGGGTCATCTGGATGTCCGATCCGCC 3’

RHuVκ2aBACKFv: 5’ GGAGACTGAGTCATCACAACATCCGATCCGCC 3’

RHuVκ3aBACKFv: 5’ GGAGACTGCGTCAACACAATTTCCGATCCGCC 3’

RHuVκ4aBACKFv: 5’ GGAGACTGGGTCATCACGATGTCCGATCCGCC 3’

RHuVκ5aBACKFv: 5’ GGAGACTGCGTGAGTGTCGTTTCCGATCCGCC 3’

RHuVκ6aBACKFv: 5’ GGAGACTGAGTCAGCACAATTTCCGATCCGCC 3’

RHuVλBACK1Fv: 5’ GGCGGCTGCGTCAACACAGACTGCGATCCGCCACCGCCAGAG 3’

RHuVλBACK2Fv: 5’ GCAGGCTGAGTCAGAGCAGACTGCGATCCGCCACCGCCAGAG 3’

RHuVλBACK3aFv: 5’ GGTGGCTGAGTCAGCACATAGGACGATCCGCCACCGCCAGAG 3’

RHuVλBACK3bFv: 5’ GGGTCCTGAGTCAGCTCAGAAGACGATCCGCCACCGCCAGAG 3’

RHuVλBACK4Fv: 5’ GGCGGTTGAGTCAGTATAACGTGCGATCCGCCACCGCCAGAG 3’

RHuVλBACK5Fv: 5’ GACGGCTGAGTCAGCACAGACTGCGATCCGCCACCGCCAGAG 3’

RHuVλBACK6Fv: 5’ TGGGGCTGAGTCAGCATAAAATTCGATCCGCCACCGCCAGAG 3’

DNAse treatment of RNA

DNAse treatment of RNA sample was performed by using DNAse (Merck, #AMPD1-1KT) according to the protocol on detailed in Suppl.Table S5

Reaction mix for DNAsi treatment	
RNA	5 µg
10X Buffer	1X
DNAsi 1 U/µl	1 µl
H20 DEPC	Q.S.
RT per 15 min	
Stop solution 50 mM EDTA	1µl
Tot	11 µl
70°C per 10 min-> 4°C	

Suppl. Table S5. DNAse treatment protocol.

RNA Retrotranscription to cDNA

Retrotrascription of RNA to obtain cDNA IgM or IgG/IgA specific was performed using AMV Reverse Transcriptase (Promega, #M510F) according to the protocols detailed in Suppl.Table S6. The primers used for IgM cDNA and IgG/IgA cDNA are different.

Reaction mix for retrotrscription cDNA for IgM library	
RNA DNAsi treated	5 µg
5X AMV Buffer	1X
100 mM DTT	10 mM
10 mM dNTPs	0,25 mM
Primer HuIgMFOR 10 µM	0,4 mM
Primer HuCκFOR 10 µM	0,4 mM
Primer HuCλFOR 10 µM	0,4 mM
RNAsin 40 U/µl	80 units
AMV 10 U/µl	25 units
H20 DEPC	Q.S.
FINAL VOLUME	50 µl
42°C per 1h<br>99°C per 3 min<br>4°C per ∞	

Reaction mix for retrotrscription cDNA for IgG/A library	
RNA DNAsi treated	5 µg
5X AMV Buffer	1X
100 mM DTT	10 mM
10 mM dNTPs	0,25 mM
Primer HuIgG1-4CH1FOR 10 µM	0,4 mM
Primer hIGA_r1 10 µM	0,4 mM
Primer HuCκFOR 10 µM	0,4 mM
Primer HuCλFOR 10 µM	0,4 mM
RNAsin 40 U/µl	80 units
AMV 10 U/µl	25 units
H20 DEPC	Q.S.
FINAL VOLUME	50 µl

Suppl. Table S6. Retrotranscription protocols for IgM cDNA (left) and for IgG/IgA cDNA (right) used.

PCRs

High Fidelity Q5 polymerase (New England Biolabs, # M0491L) was used in the crucial PCR steps to create the recombinant antibody domain scFv library.

The annealing temperature (TA) was chosen case-by-case according to the primer sequence, template overlap and reaction parameters, using the dedicated function of the bioinformatic tool SnapGene. The extension time was calculated assuming for the Q5 polymerase an elongation rate of about 1000 nt/min.

cDNA of IgM and IgG/IgA and (heavy and light chains) was used as template to amplify VH and VL (both kappa and lambda, henceforth indicated as VK and Vλ) regions. Primers were designed to anneal to the external framework regions of the V genes and include restriction sites for BssHII at the 5' end, and NheI at the 3' end detailed in Suppl. Table S7.

**PCR reaction mix for VHs**	
5X Buffer Q5	1X
10 mM dNTPs	0,2 mM
Mix 6 Primer BssHII-HuVHsBACK 10 µM each	0,5 µM each
Mix 4 Primer HuJHsFOR 10 µM each	0,5 µM each
Q5 polymerase 2 U/µl	1 unit
H20	Q.S.
cDNA	500 ng
FINAL VOLUME	50 µl

**PCR reaction mix for VKs**	
5X Buffer Q5	1X
10 mM dNTPs	0,2 mM
Mix 6 Primer HuVκsBACK 10 µM each	0,5 µM each
Mix 5 Primer NheI-HuJκsFOR 10 µM each	0,5 µM each
Q5 polymerase 2 U/µl	1 unit
H20	Q.S.
cDNA	500 ng
FINAL VOLUME	50 µl

**PCR reaction mix for Vλs**	
5X Buffer Q5	1X
10 mM dNTPs	0,2 mM
Mix 7 Primer HuVλsBACK 10 µM each	0,5 µM each
Mix 3 Primer NheI-HuJλsFOR 10 µM each	0,5 µM each
Q5 polymerase 2 U/µl	1 unit
H20	Q.S.
cDNA	500 ng
FINAL VOLUME	50 µl

98°C x 1 min	
98°C x 10 sec	X 25 cycles
60°C x 30 sec	
72°C x 45 sec	
72°Cx 3 min	
12°C ∞	

Suppl.Table S7. PCR protocols used to amplify VHs,VKs, Vλs.

The amplified VHs, VKs and Vλs were run on a 1% Agarose gel and purified with Wizard® SV Gel and PCR Clean-Up System (Promega, # A9282).

(G4S)3 linker construction.

A 45 bp linker (G4S)3 was amplified from a pre-existing plasmid using a set of primers, such that a linker (G4S)3 had 5' specific for VHs and 3' blunt, whereas 2 linkers (G4S)3 had 3' specific for VKs or Vλs and 5' blunt (Suppl.Table S8).

**PCR reaction mix for VHs-Linker BLUNT**	
5X Buffer Q5	1X
10 mM dNTPs	0,2 mM
Mix 4 Primer RHuJHs 10 µM each	0,5 µM each
Primer MinusLinker(G4S)3 10 µM each	0,5 µM each
Q5 polymerase 2 U/µl	1 unit
H20	Q.S.
pL220 MintV1 1 ng/µl	1 ng
FINAL VOLUME	50 µl

**PCR reaction mix for Linker-VKs BLUNT**	
5X Buffer Q5	1X
10 mM dNTPs	0,2 mM
Mix 6 Primer RHuVκsBACKFv 10µM each	0,5 µM each
Primer PlusLinker(G4S)3 10µM each	0,5 µM each
Q5 polymerase 2 U/µl	1 unit
H20	Q.S.
pL220 MintV1 1 ng/µl	1 ng
FINAL VOLUME	50 µl

98°C x 30 sec	
98°C x 10 sec	X 25 - 35 cycles
65°C - 69°C x 30 sec	
72°C x 30 sec	
72°C x 30 smin	
12°C ∞	

**PCR reaction mix for Linker-Vλs BLUNT**	
5X Buffer Q5	1X
10 mM dNTPs	0,2 mM
Mix 7 Primer RHuVλsBACKFv 10µM each	0,5 µM each
Primer PlusLinker(G4S)3 10µM each	0,5 µM each
Q5 polymerase 2 U/µl	1 unit
H20	Q.S.
pL220 MintV1 1 ng/µl	1 ng
FINAL VOLUME	50 µl

Suppl.Table S8. PCR protocols used to amplify linker for VHs,VKs, Vλs.

The amplified Linkers for VHs, VKs and Vλs were run on a 2% Agarose gel and purified with Wizard® SV Gel and PCR Clean-Up System.

The mix of the different classes of VHs was used in the third step in a PCR to join VH products to VH specific linker forming BLOCK A (BssHII-VHs-Linker Blunt) (Suppl.Table S9, left panel). Similarly, PCRs were performed to join, respectively, VK and Vλ products to VK and Vλ specific linkers (Suppl.Table S9, center and right panel).

PCR reaction mix for BLOCK A BssHII-VHs-Linker Blunt	
5x Buffer Q5	1x
10 mM dNTPs	0,2 mM
Mix 6 Primer BssHII-HuVHsBACK 10 µM each	0,5 µM each
Primer MinusLinker(G4S)3 10 µM each	0,5 µM each
Q5 polymerase 2 U/µl	1 unit
H20	Q.S.
VHs library # dated	100 ng
Linker x VHs dated	100 ng
FINAL VOLUME	50 µl

PCR reaction mix for BLOCK B Linker Blunt-VKs-NheI	
5x Buffer Q5	1x
10 mM dNTPs	0,2 mM
Mix 5 Primer NheI-HuJκsFOR 10 µM each	0,5 µM each
Primer PlusLinker(G4S)3 10 µM each	0,5 µM each
Q5 polymerase 2 U/µl	1 unit
H20	Q.S.
VKs library # dated	100 ng
Linker x VHs dated	100 ng
FINAL VOLUME	50 µl

PCR reaction mix for BLOCK C Linker Blunt-Vλs-NheI	
5x Buffer Q5	1x
10 mM dNTPs	0,2 mM
Mix 3 Primer NheI-HuJλsFOR 10 µM each	0,5 µM each
Primer PlusLinker(G4S)3 10 µM each	0,5 µM each
Q5 polymerase 2 U/µl	1 unit
H20	Q.S.
Vλs library # dated	100 ng
Linker x VHs dated	100 ng
FINAL VOLUME	50 µl

98°C x 30 sec	
98°C x 10 sec	X 25 cycles
60°C x 30 sec	
72°C x 30 min	
72°C x 5 min	
12°C ∞	

Suppl.Table S9. PCR protocols used to amplify Blocks.

The amplified BLOCKs (A, B and C) were run on a 1% Agarose gel and purified with Wizard® SV Gel and PCR Clean-Up System

Pullthrough of “blocks” and insertion of restriction sites for BssHII at the 5’ end, and NheI at the 3’ end, were performed using Vent DNA Polymerase according to the following protocols (Suppl. Table S10).

In this step the first 10 cycles were run without primers allowing the joining of the different blocks, using the overlapping linker (G4S)3 sequence for priming. Then, primers used for the 5’ of VHs bearing restriction site for BssHII and primers used for the 3’ of VKs bearing restriction site for NheI were added to the mix, generating the final ~1000 bp scFvs products called Assembly 1 (BssHII-VHs-Linker-VKs-NheI). Similarly, a pull through PCR to join VH blocks and Vλ blocks was performed to obtain the scFv products called Assembly 2 (BssHII-VHs-Linker-VKs-NheI).

The amplified Assembly 1 and 2 were run on a 1% Agarose gel and purified with Qiagen MinElute Gel Extraction Kit (Qiagen, # 28604).

PCR reaction mix for Assembly 1 BssHII-VHs-Linker-VKs-NheI	
10x Buffer Vent	1x
10 mM dNTPs	0,2 mM
Vent polymerase 2 U/µl	1 unit
H20	Q.S.
BLOCK A BssHII-VHs-Linker Blunt from library # dated	100 ng
BLOCK B Linker Blunt-VKs-NheI from library # dated	100 ng
Tot	50 µl
Add after the first 10 cycles	
Mix 6 Primer BssHII-HuVHsBACK 50 µM each	1,15 µM
Mix 5 Primer NheI-HuJκsFOR 50 µM each	1,15 µM
Tot	52,4 µl each reaction

95°C x 5 min	
95°C x 30 sec	X 10 cycles
67°C x 1 min	
72°C x 1 min	
95°C x 1 min	Pause, add primers
95°C x 30 sec	X 10 cycles
67°C x 1 min	
72°C x 1 min	
72°C x 5 min	
12°C x ∞	

PCR reaction mix for Assembly 2 BssHII-VHs-Linker-VKs-NheI	
10x Buffer Vent	1x
10 mM dNTPs	0,2 mM
Vent polymerase 2 U/µl	1 unit
H20	Q.S.
BLOCK A BssHII-VHs-Linker Blunt from library # dated	100 ng
BLOCK C Linker Blunt-Vλs-NheI from library # dated	100 ng
Tot	50 µl
Add after the first 10 cycles	
Mix 6 Primer BssHII-HuVHsBACK 50 µM each	1,15 µM
Mix 5 Primer NheI-HuJλsFOR 50 µM each	1,15 µM
Tot	52,4 µl each reaction

Suppl. Table S10. Pull through PCR protocol used in this work.

Gel electrophoresis and purification

PCR amplification products of every step of library construction were resolved by agarose gel electrophoresis stained with ethidium bromide (EtBr) to visualise PCR products and the bands of the expected molecular weight were cut and purified using either Wizard® SV Gel and PCR Clean-Up System (Promega) or Qiagen MinElute Gel Extraction Kit as indicated in the PCR protocols.

All DNA quantifications were done by using Nanodrop spectrophotometry.

DNA digestion

The enzymatic digestion of the scFvs produced (inserts) and of the pLinker220 (vector) was performed incubating the DNA (1-2 ug) with NheI (10 U/µl) (New England Biolab cat.# R0131L) for 2h 30 min at 37˚C, then BssHII (5 U/µl) (New England Biolab cat.# R0199L) was added and the reaction incubated again for 2h 30 min at 50˚C. Digestion of the vector was followed by Calf Intestinal alkaline Phosphatase (CIP) treatment to remove 5'-phosphate groups from the vector, to prevent

self-ligation. This property greatly reduces background (plasmids without insert) in cloning procedures. CIP was added and the reaction was incubated at 37°C for 1h. (Supp.Table S11).

Variable quantities of DNA, depending on the usage of the digested product, were used, typically in the range of 1 – 2 μg for the reaction volumes here reported. The quantity of restriction enzymes was calculated on the base of the DNA concentration, however always maintaining the concentration of the glycerol, in which the enzymes were stored, below 40% of the final volume. The digested products were purified using the MinElute Gel extraction kit (Qiagen).

BUFFER CutSMART 10X	1X
RESTRICTION ENZYME(S)	1,0 – 2,0 μl for each enzyme
DNA Variable	typically 1,0 – 2,0 μg
CIP	1,0 μl
H2O	Q.S.
FINAL VOLUME	60 μl

Suppl.Table S11. Reaction mix used for the DNA enzymatic digestion.

DNA ligation

The ligation reaction between the BssHII/NheI digested pLinker220 vectors and the BssHII/NheI digested scFvs was performed using the following reaction mix, incubated overnight (ON) at 16°C (Suppl.Table S12).

The required amounts of DNA were calculated, considering the number of base pair (bp) of both the DNA vector and insert, according to the following formula, typically using 2000 ng of DNA vector:

ng Insert =(ng Vector × bp Insert)/(bp Vector)×3

As negative control, a ligation reaction with the vector alone was set up in parallel.

BUFFER T4 10X	1X
VECTOR	Variable (1200-2000 ng)
INSERT(S)	Variable (350-600 ng)
T4 LIGASE (400 U/μl)	400 units
H2O	Q.S.
FINAL VOLUME	60 μl

Suppl.Table S12. Reaction mix used for the DNA ligation.

Small scale bacterial transformation of library ligation by electroporation

As a crucial step of molecular cloning, competent bacteria were transformed with the ligation products to amplify the new cloned plasmids. E-Max Efficiency E. coli DH5α were used as electrocompetent cells and each aliquot of bacterial cells was electroporated with 250 ng of the ligation product or 250 ng of the digested empty vector used for the ligation to evaluate the background (number of empty vectors that were not correctly digested).

The electroporated cells were, therefore, inoculated in SOC medium (950 µl for each aliquot of cells) and left for one hour to recover in a shaker incubator at 37°C. After this step, serial dilutions of the transformation were plated onto LB plates with ampicillin and incubated at 37°C overnight to verify transformation efficiency. The result of this small-scale transformation was evaluated to be used for the big scale transformation.

Big scale transformation of library ligation

According to the efficiency obtained in the small scale transformation, multiple transformations for each library ligation (each with 250 ng) were performed to obtain around 1 million cells to be inoculated into 1 L of LB + Sea Prep Agar + ampicillin, as described in Elsaesser and Paysan[7]. After the inoculum of the recovered cells, 1 ml was collected to be plated with serial dilutions to verify the big scale transformation efficiency. The 1 L of LB + Sea Prep Agar + ampicillin with the inoculated transformed bacterial cells in Sea Prep Agar was poured in pre-chilled sterile stainless steel container (approximately 200 × 300 × 50 mm3; Neolab, Heidelberg, Germany) on wet ice in a cold room and left on ice at 4°C for 1 hour and 30 min, and then transferred to an incubator at 37°C for 40 hours.

The visible spherical bacterial colonies embedded in the semi-liquid gel were collected by centrifugation at 8000 g for 20 min at room temperature. The pellet was washed with 100 mL of LB medium and centrifuged again at 8000 g for 20 min at room temperature. Plasmid DNA from the pellet was extracted using Qiagen GIGAprep kit, according to the manufacturer’s instructions.

NGS LIBRRAY SEQUENCING

ID PRIMER	REGION	SEQUENCE
VH_fus_F	VH	CTATGGGCAGTCGGTGATCAGCCGAGCGCGCATGCC
VH_fus_R	VH	TGCGTGTCTCCGACTCAGGATANNNNNNNNNNNNNGCCTGAACCGCCTCCACC
VL_fus_F	VL	CTATGGGCAGTCGGTGATGAGGTGGCTCTGGCGGT
VL_fus_R_B01	VL	TGCGTGTCTCCGACTCAGATTAGATANNNNNNNNNNNNNCGGAGCTCGAAACGCTAGC
VL_fus_R_B02	VL	TGCGTGTCTCCGACTCAGTCTCGATANNNNNNNNNNNNNCGGAGCTCGAAACGCTAGC
VL_fus_R_B03	VL	TGCGTGTCTCCGACTCAGTTACGATANNNNNNNNNNNNNCGGAGCTCGAAACGCTAGC

Suppl.Table S13. Primers used in the first PCR to amplify VH and VL for sequencing

Green: sequence complementary to the trP1_fus primer; Purple: sequence complementary to the A_fus primer; Blu: sequence to identify UMI; Red: barcode; Black: sequence complementary to the linker between VH and VL; Orange: sequence complementary to the pLinker220 plasmid region flanking the VH or VL

ID PRIMER	SEQUENCE
A_fus	CCATCTCATCCCTGCGTGTCTCCGACTCAG
trP1_fus	CCTCTCTATGGGCAGTCGGTGAT

Suppl.Table S14. Primers used in the second PCR to amplify VH and VL for sequencing

Purple: sequence complementary to the purple sequence of primer used in the first PCR reaction
Green: sequence complementary to the green sequence of primer used in the first PCR reaction

Anti Nucleocapsid scFvs purification from *E. Coli*

Purification of scFv 5

The purification of scFv 5 was achieved by cation exchange chromatography. The profile of elution is shown in Suppl. Fig S13.

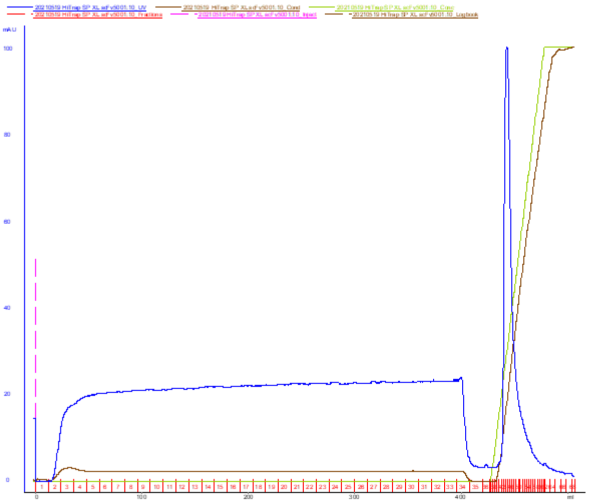

Suppl. Fig S13. Purification of scFv 5: a representative chromatogram of the cation exchange chromatography.

Purification of scFv 24

The purification of scFv 24 was carried out by cation exchange chromatography. The resulting chromatogram is shown in Suppl. Fig S14.

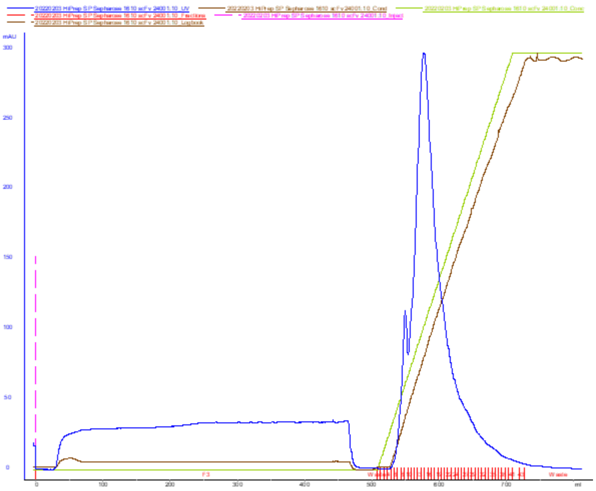

Suppl. Fig S14. Purification of scFv 24: a representative chromatogram of the cation exchange chromatography.

The fractions were analyzed by SDS-PAGE. The analysis showed that (Suppl. Fig S15b) the scFv was present in all the analyzed fractions but in presence of contaminants of different molecular weights. Then the fractions were collected into three pools A, B and C, as indicated in Suppl. Fig Sa.15a.

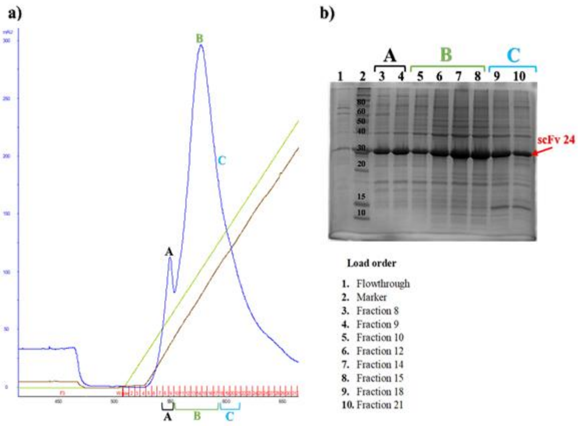

Suppl. Fig S15. (a) detail of the elution profile of the scFv 24 illustrating the fractions collected – pool A: fractions 8-9, pool B: fractions 10-17, pool C: fractions 18-21. **(b)** A representative SDS-PAGE of the fractions from the cation exchange chromatography of scFv 24.

Purification of scFv 77

Purification of scFv 77 was achieved by cation exchange chromatography. As shown in Suppl. Fig S16, the profile of elution is composed of 3 unresolved main peaks.

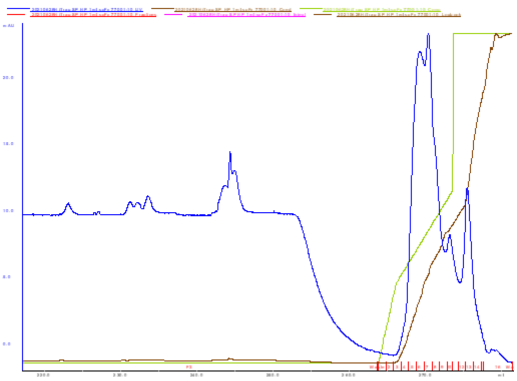

Suppl. Fig S16. Purification of scFv 77: a representative chromatogram of the cation exchange chromatography.

Purification of scFv 93

The purification of scFv 93 was achieved by anion exchange chromatography. The chromatogram (Suppl. Fig S17) was composed of three peaks.

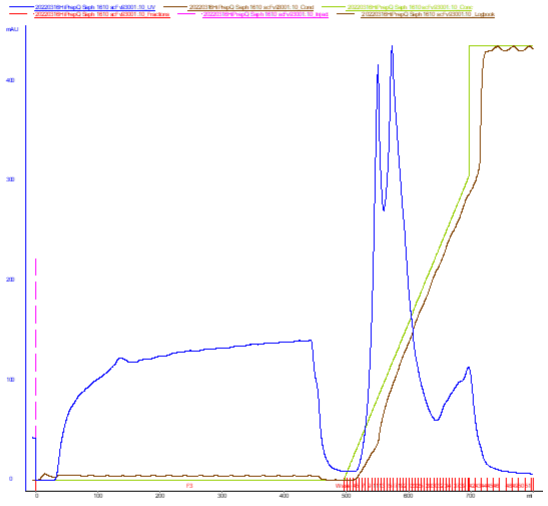

Suppl. Fig S17. Purification of scFv 93: a representative chromatogram of the anion exchange chromatography.

The fractions corresponding to the peaks were analyzed by SDS-PAGE. As evident from Suppl. Fig S18b, a band corresponding to the scFv was present in all the analyzed fractions.

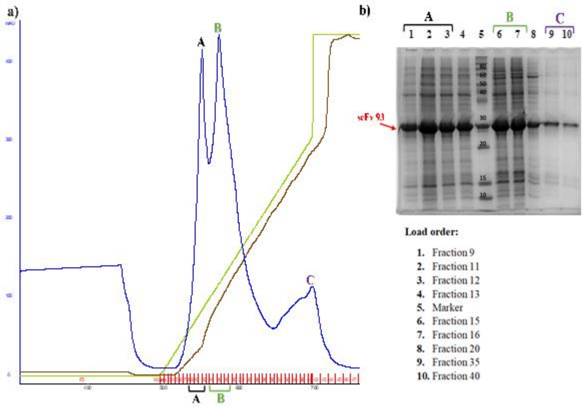

Suppl. Fig S18. (a) Detail of the elution profile of the scFv 93 indicating the fractions collected – pool A: fractions 9-12, pool B: fractions 14-19. **(b)** A representative SDS-PAGE of the fractions from the purification of scFv 93.

Pools A and B were analyzed by analytical gel filtration (see Suppl. Fig. S18). Pool C was not subjected to gel filtration because of the presence of contaminant nucleic acid in the sample, as shown by the spectrophotometric analysis (data not shown).

Purification of scFv 255

The purification of scFv 255 was achieved by anion exchange chromatography. The chromatogram was characterized by two unresolved peaks (Suppl. Fig S19).

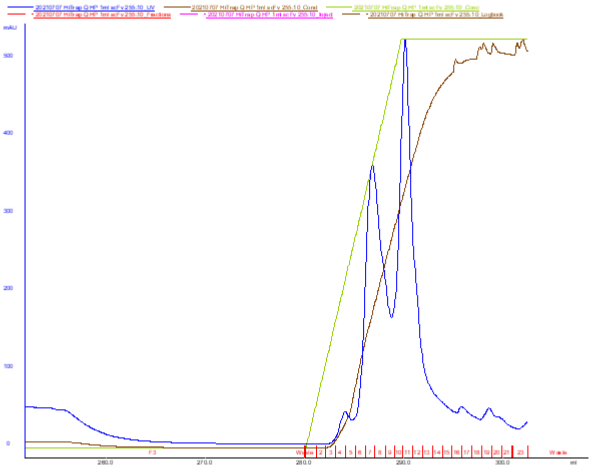

Suppl. Fig S19. Purification of scFv 255: a representative chromatogram of the anion exchange chromatography.

Purification of scFv 261

The purification of scFv 261 was achieved by cation exchange chromatography (Suppl. Fig S20).

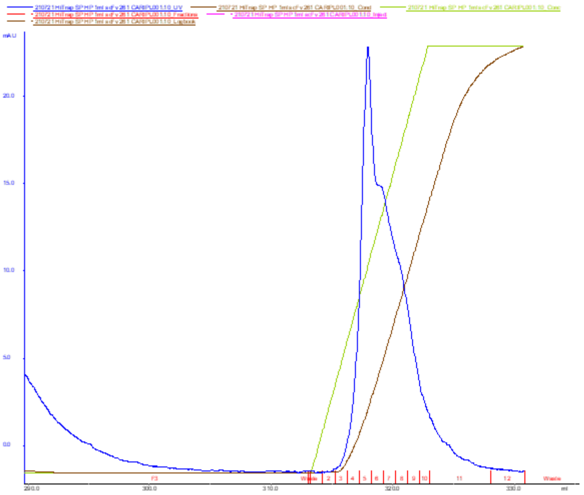

Suppl. Fig S20. Purification of scFv 261: a representative chromatogram of the cation exchange chromatography.

5.1.8 Purification of scFv 291

The purification of scFv 291 was carried out by cation exchange chromatography. (Suppl. Fig S21).

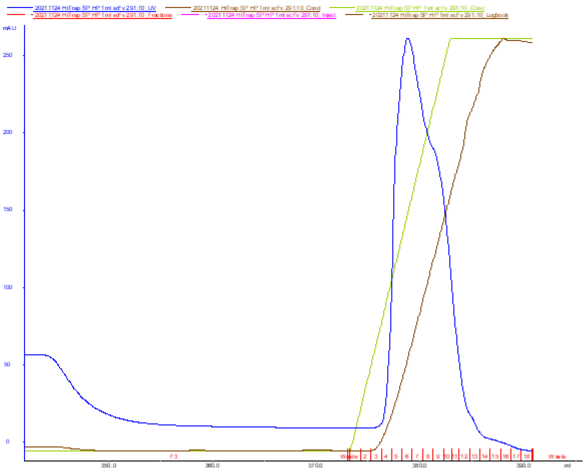

Suppl. Fig S21. Purification of scFv 291: a representative chromatogram of the cation exchange chromatography.

Purification of scFv 336

The purification of scFv 336 was carried out by cation exchange chromatography. The chromatogram showed two peaks (Suppl. Fig S22).

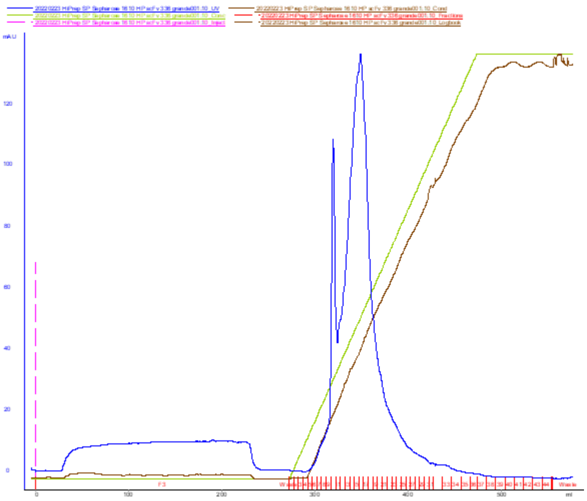

Suppl. Fig S22. Purification of scFv 336: a representative chromatogram of the cation exchange chromatography.

The fractions corresponding to the peaks were analyzed by SDS-PAGE. The analysis showed that (Suppl. Fig S23b) the scFv was present in all the analyzed fractions, but in presence of contaminants. For each peak, the fractions having higher concentrations of scFv were pooled, as indicated in Suppl. Fig. S23a.

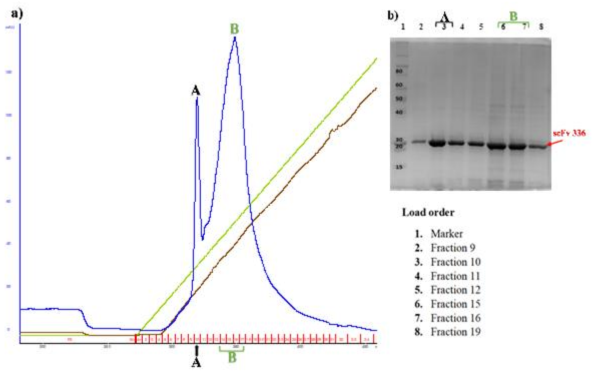

Suppl. Fig S23. (a) Detail of the elution profile of the cation exchange chromatography illustrating the fractions collected – pool A: fraction 10, pool B: fractions 14-17. **(b)** A representative SDS-PAGE of the fractions of the purification of scFv 336.

**Forward_ N-FL**	5’-TCGGCCATGGGCTCCGATAACG-3’
**Reverse_ N-FL**	5’-ATAGTTTACGCCGGCGAATTCGAACC-3’
**Forward-1_N-CTD**	5’-ACCAAAAAATCAGCGGCAGAAGCATC-3’
**Reverse-1_ N-CTD**	5’-GCCCTGAAAATAAAGATTCTCGCTCATG-3’
**Forward-2_N-CTD**	5’-TAAGCGGCCGCGAGCTCGGAT-3’
**Reverse-2_ N-CTD**	5’-AGGAAATGTTTTGTAAGCATCAATATGCTTGTTGAG-3’
**Forward_AT-N-CTD**	5’-GCACAAAAAATTGAATGGCATGAGACCAAAAAATCAGCGGCAGAAG-3’
**Reverse_AT-N-CTD**	5’-TTCGAAAATGTCGTTGAGTCCGCCCTGAAAATAAAGATTCTC-3’

Suppl. Table S15. Primers for cloning of the N-protein constructs

IMAGES of the ORIGINAL WESTERN BLOTS (not cropped)

B

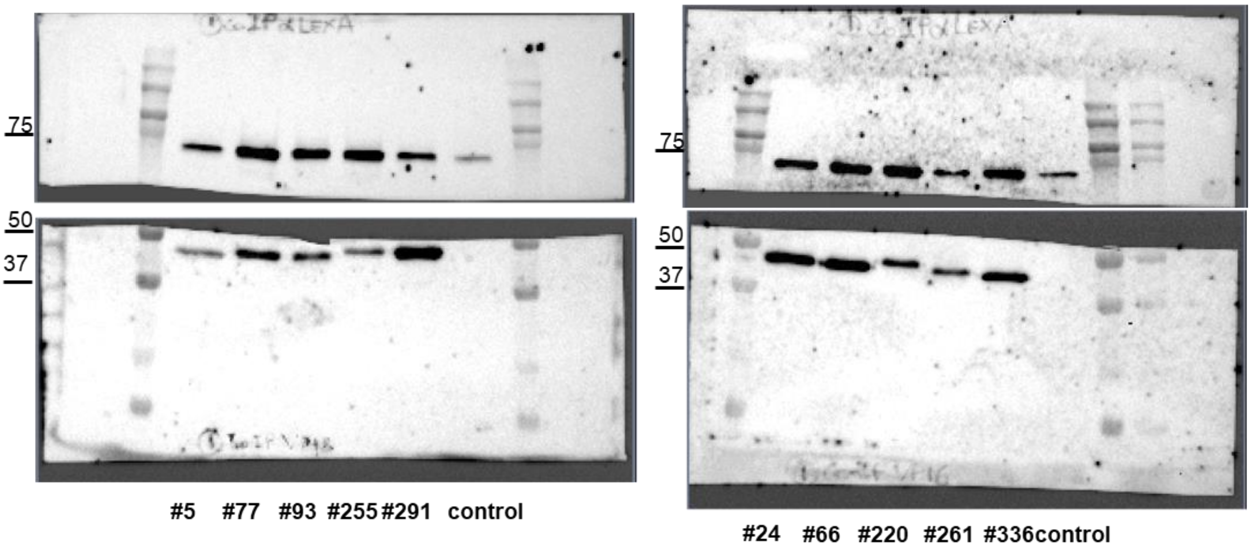

C

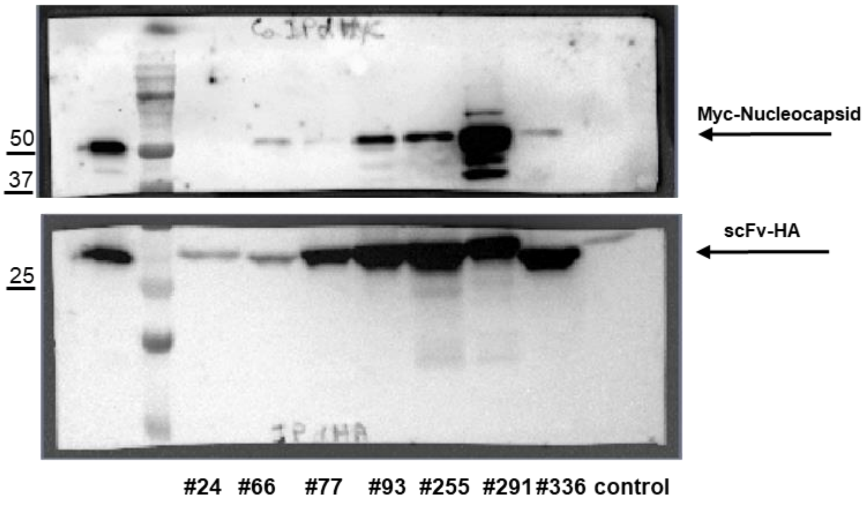

Fig. 1. Co-immunoprecipitation of Nucleocapsid full length protein with scFv anti Nucleocapsid in yeast (B) and in HEK 293T cells (C).

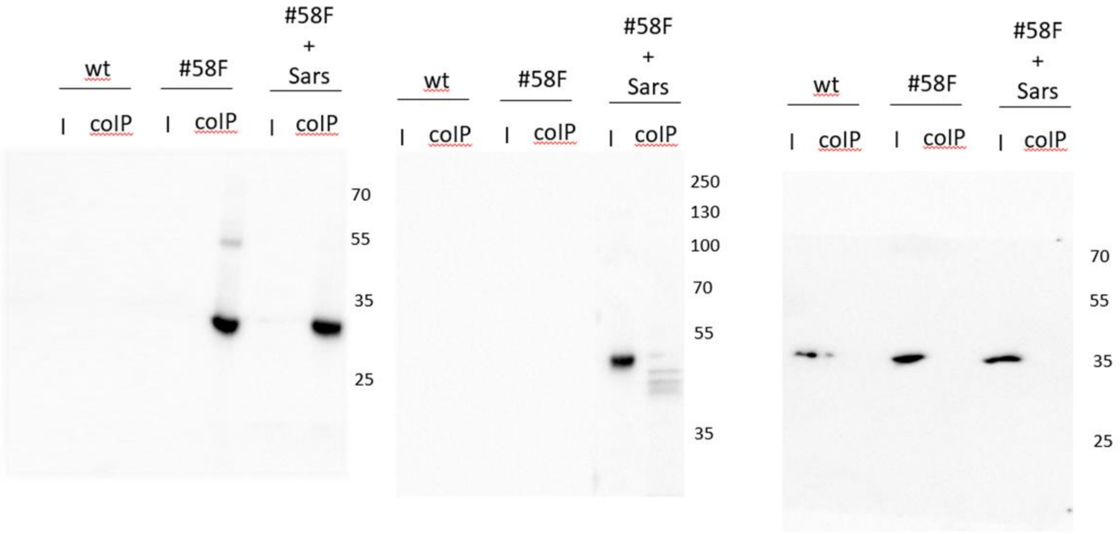

Fig. 5-A. Co-immunoprecipitation of SARS-CoV 2 viral N protein with anti N scFvs expressed in Vero TMPRSS2 cells.

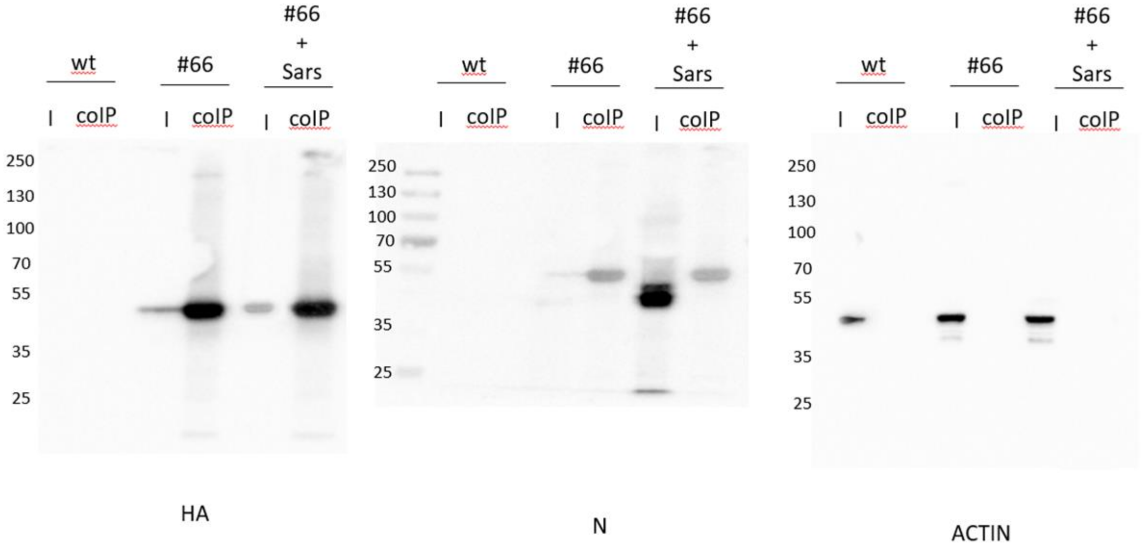

Fig. 5 B. Co-immunoprecipitation of SARS-CoV 2 viral N protein with anti N scFvs expressed in Vero TMPRSS2 cells.

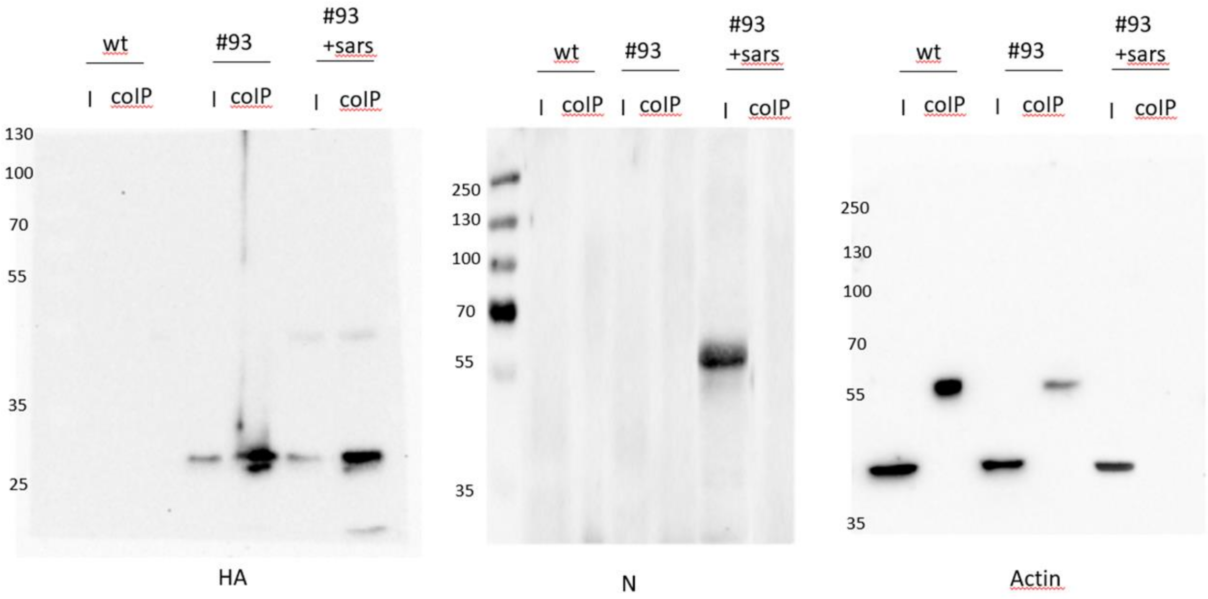

Fig. 5 C. Co-immunoprecipitation of SARS-CoV 2 viral N protein with anti N scFvs expressed in Vero TMPRSS2 cells.

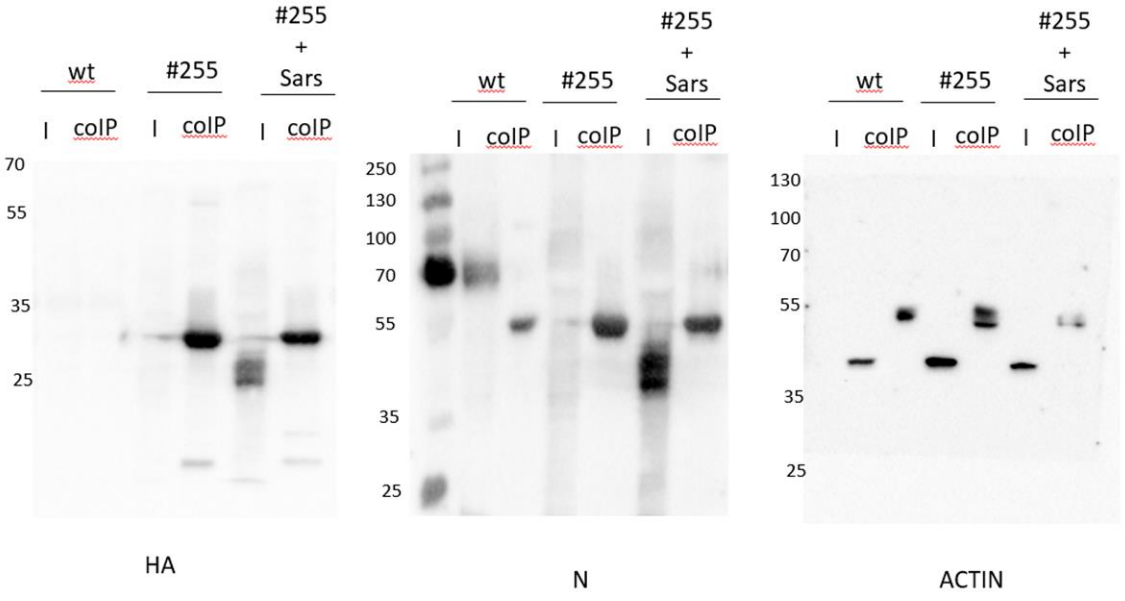

Fig. 5 D. Co-immunoprecipitation of SARS-CoV 2 viral N protein with anti N scFvs expressed in Vero TMPRSS2 cells.

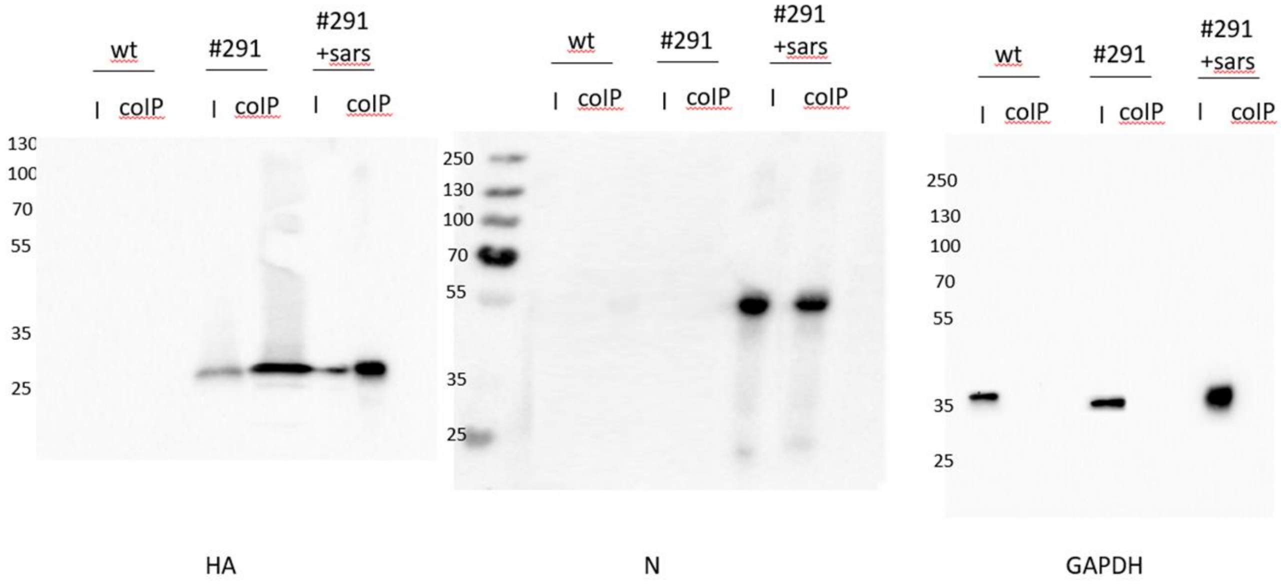

Fig. 5 E. Co-immunoprecipitation of SARS-CoV 2 viral N protein with anti N scFvs expressed in Vero TMPRSS2 cells.

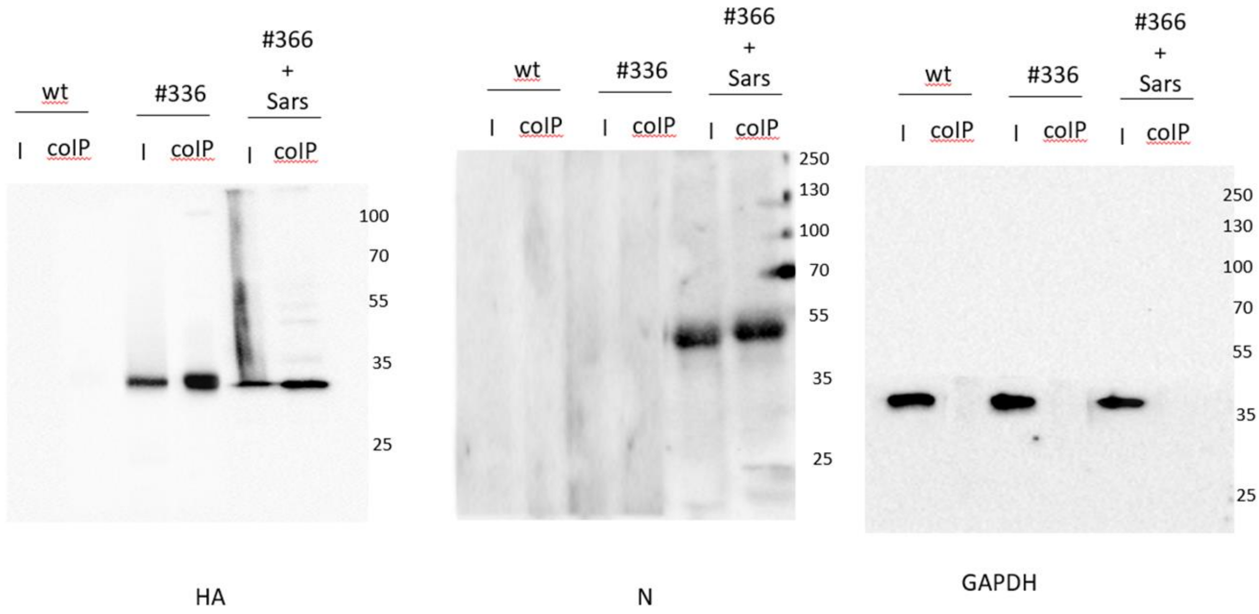

Fig. 5 F. Co-immunoprecipitation of SARS-CoV 2 viral N protein with anti N scFvs expressed in Vero TMPRSS2 cells.

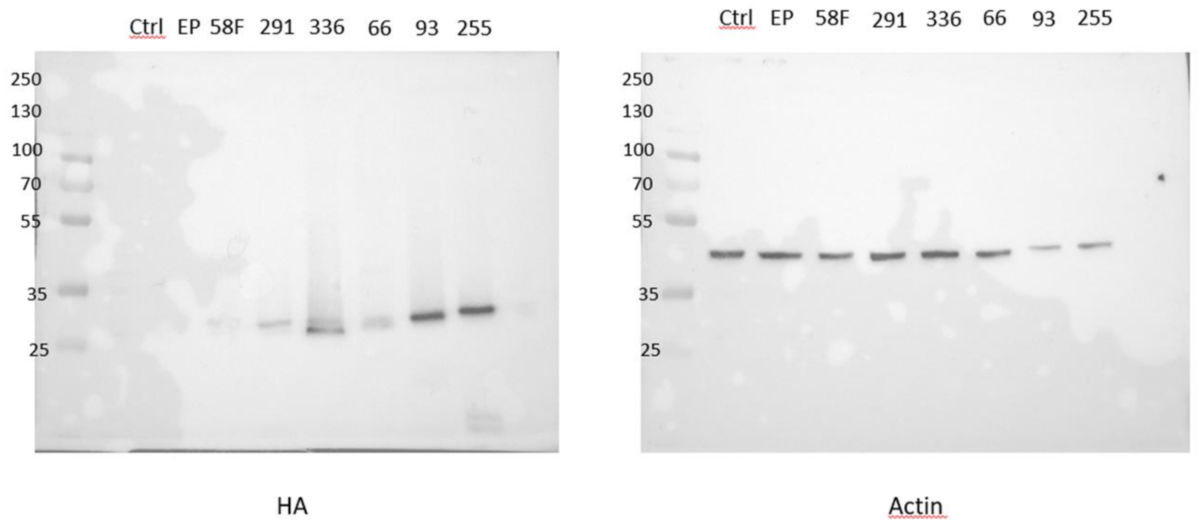

Suppl. Fig.S11B. Vero E6 cell extracts expressing the scFvs-HA (28 KDa) and an unrelated scFv (#58F).

REFERENCES

1. Gangavarapu, K. *et al.* Outbreak.info genomic reports: scalable and dynamic surveillance of SARS-CoV-2 variants and mutations. *Nat. Methods* **20**, 512 (2023).
2. Khare, S. *et al.* GISAID's Role in Pandemic Response. *China CDC Wkly.* **3**, 1049 (2021).
3. Kreuzberger, N. *et al.* SARS-CoV-2-neutralising monoclonal antibodies for treatment of COVID-19. *Cochrane Database Syst. Rev.* **2021**, (2021).
4. Marks, J. D. & Bradbury, A. PCR cloning of human immunoglobulin genes. *Methods Mol. Biol.* **248**, 117–134 (2004).
5. Fantini, M. *et al.* Assessment of antibody library diversity through next generation sequencing and technical error compensation. *PLoS One* **12**, (2017).
6. Visintin, M., Meli, G. A., Cannistraci, I. & Cattaneo, A. Intracellular antibodies for proteomics. *J Immunol Methods* **290**, 135–153 (2004).
7. Elsaesser, R. & Paysan, J. Liquid gel amplification of complex plasmid libraries. *Biotechniques* **37**, 200,202 (2004).
